# From Frequency Domain to Time Transient Methods for Halide Perovskite Solar Cells: The Connections of IMPS, IMVS, TPC, and TPV

**DOI:** 10.1021/acs.jpclett.1c02065

**Published:** 2021-08-13

**Authors:** Juan Bisquert, Mathijs Janssen

**Affiliations:** † Institute of Advanced Materials (INAM), Universitat Jaume I, 12006 Castelló, Spain; ‡ Department of Mathematics, Mechanics Division, University of Oslo, N-0851 Oslo, Norway

## Abstract

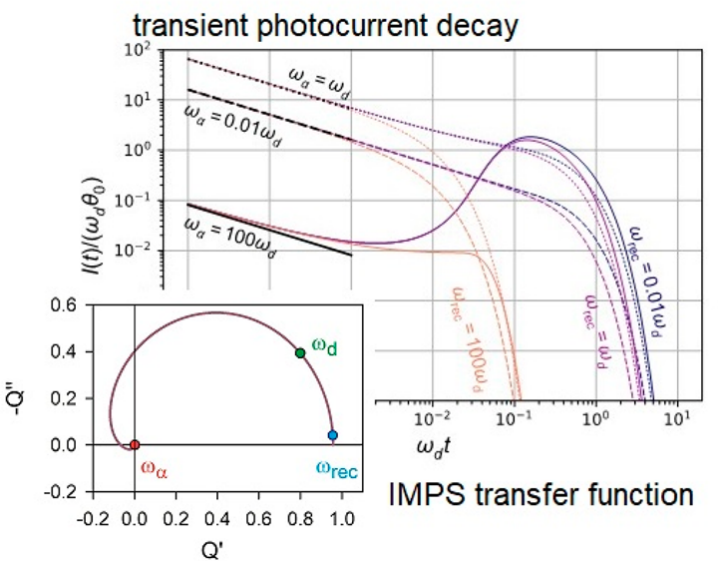

The correlation of different methods of measurement can become an important tool to identify the dominant physical elements that govern the electronic and ionic dynamics in perovskite solar cells. The diverse phenomena underlying the response of halide perovskite materials to different stimuli are reflected in time-domain measurements, where transients appear with time scales spanning orders of magnitude, from nanoseconds to hours. We discuss the connection between different frequency- and time-domain methods to probe the voltage and current response of halide perovskite solar cells to different small perturbations. To solve the frequency-to-time transformation, we start from models of the transfer function of intensity-modulated photocurrent spectroscopy (IMPS) and derive the associated impulse response function, the transient photocurrent (TPC), in response to a short light pulse. Similarly, we determine the transient photovoltage (TPV) starting from the intensity-modulated photovoltage spectroscopy (IMVS) transfer function. We also discuss the open-circuit voltage decays (OCVD). We first show the response of simple equivalent circuit models, and then we treat the full model for generation–diffusion–recombination of electrons that shows a spiraling loop in IMPS. This model gives rise to overshoots in the time domain.

Both frequency- and time-domain techniques are major tools to understand the physical phenomena governing the behavior of halide perovskite solar cells (PSCs).^1^ Often used frequency-domain techniques include impedance spectroscopy^2^ (IS) and the light-modulated techniques intensity-modulated photocurrent spectroscopy (IMPS) and intensity-modulated photovoltage spectroscopy (IMVS).^3−7^ Meanwhile, time-domain measurements of the transient photocurrent (TPC) and transient photovoltage (TPV) in response to a short light pulse have also been widely reported and applied to derive fundamental parameters such as the electron lifetime.^8−14^ The interpretation of data obtained by these methods in the area of PSCs is often very complicated, in particular by the influence of slow ionic motion that causes polarization at the interfaces. Accordingly, a number of unexpected phenomena like negative spikes in TPC and TPV^8−13^ were explained with sophisticated physical models. PSCs exhibited other anomalous behavior, like a bounce-back of the photovoltage at intermediate times^15^ in open-circuit voltage decay (OCVD) experiments^16−18^—a large-amplitude method wherein the decay of the photovoltage is measured upon switching off the light.^19^ TPC, TPV, and OCVD methods detect a current or voltage in a device with contacts. These techniques are closely related to time-resolved optical techniques such as transient absorption spectroscopy (TAS)^20−22^ and time-resolved photoluminescence (TRPL),^23,24^ which are contactless techniques not considered in this paper.

We propose a new approach to facilitate the interpretation of time-domain measurements for recorded current or voltage transients. Instead of proposing new physical models and explanations, we exploit the intrinsic correlation between time- and frequency-domain techniques. By connecting the different experimental methods with a unique underlying theory, we provide a powerful tool for obtaining a sound interpretation of results. TPC and IMPS are closely related techniques since they rely on a small perturbation over a steady state, and their dependencies in time and frequency domains are connected mathematically by a Laplace transformation. Similarly linked are TPV and IMVS. Our aim is to make these connections manifest. We take a set of dominant models that have been suggested to describe the frequency-domain light-modulated techniques, and we find the corresponding time-domain response under a short light perturbation. This approach will assist disambiguation in modeling, detect measurement artifacts, and expose the dominant physical elements that respond in different types of experiments. We will start with the simplest equivalent circuit models that are used for the interpretation of solar cells in general and PSCs specifically. Thereafter we will show the time transient shapes of PSCs under diffusion–recombination control.

*Transfer Functions in the Frequency Domain: Simple RC Circuit.* The characterization of solar cells in the mentioned techniques involves the application of three different external perturbations: voltage *V*, electrical current density *I*, and illumination flux Φ. Multiplying the latter by the elementary charge *q* yields the equivalent electrical current *q*Φ. The frequency-domain response is described by different transfer functions for impedance (*Z*), IMPS (*Q*), and IMVS (*W*). These three transfer functions are not independent but arise from the small perturbation of a general function.^25,26^ In the following, we consider small sinusoidal perturbations *V̂*(*s*), *Î*(*s*), and Φ̂(*s*) at a fixed angular frequency ω using the Laplace variable *s* = *i*ω.

We start our discussion with the parallel *R*_a_*C*_a_ circuit shown in Figure 1a. This circuit can be regarded as a basic model for a solar cell,^27,28^ in which the photogeneration occurs across the chemical capacitance *C*_a_,^29^ and the output current is reduced by the recombination resistance *R*_a_. The series resistance *R*_s_ is a realistic feature in solar cells, and here it is a necessary part of the model to avoid a short of the current generator.

**Figure 1:**
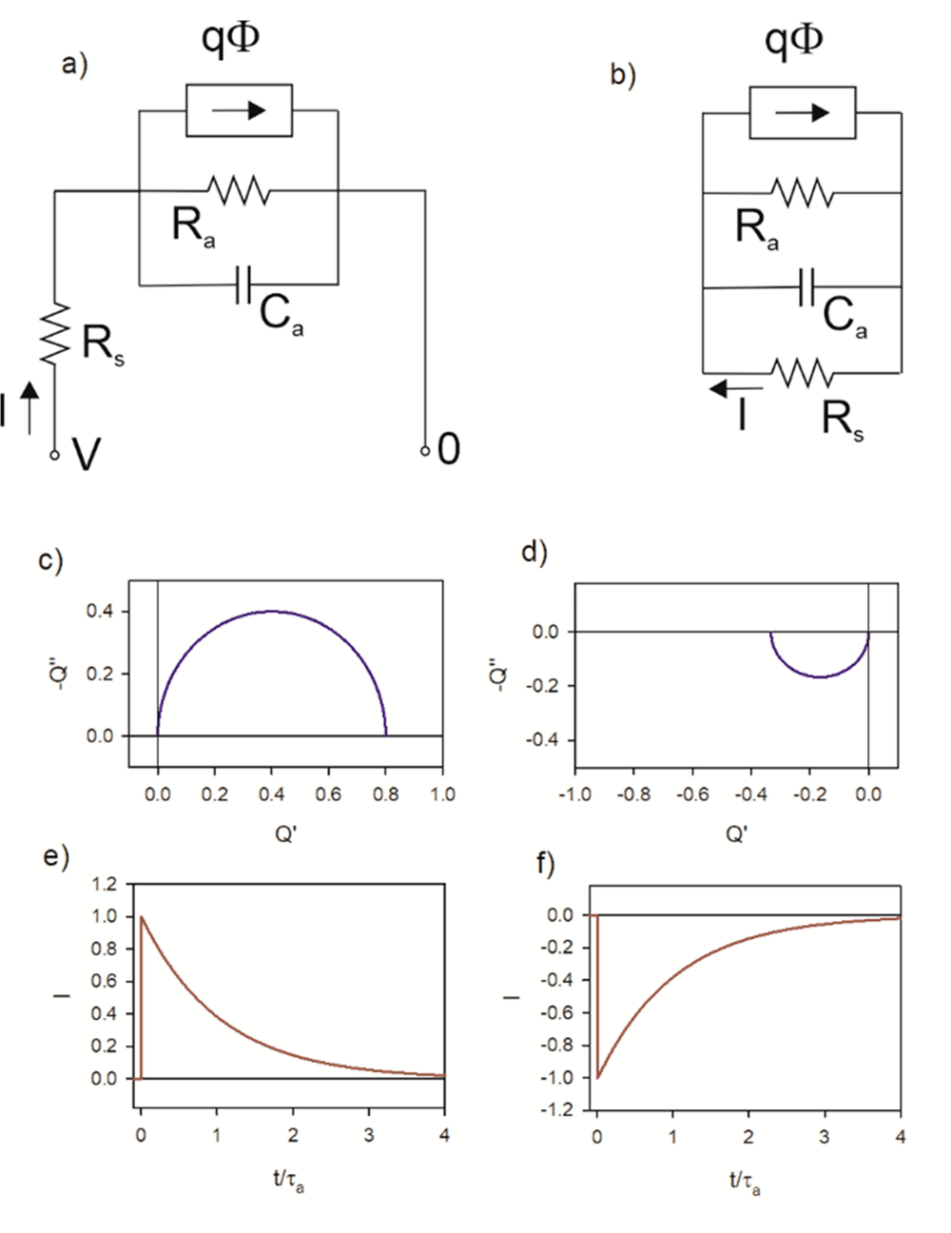
(a) RC circuit with applied light source that acts as a current source and a series resistance. The variables are small ac perturbation amplitudes. (b) The circuit in measurement of IMPS. (c, d) IMPS and (e, f) normalized TPC for the circuit of (a). (c, e) *R*_a_ = 8 Ω, *R*_s_ = 2 Ω, *C*_a_ = 0.1 *F*, τ_a_ = 0.16 s. (d, f) *R*_a_ = −2 Ω, *R*_s_ = 8 Ω, *C*_a_ = −0.1 *F*, τ_a_ = 0.26 s.

This model illustrates the methodology previously developed to obtain the different transfer functions in the frequency domain.^26^ The details of the method are explained in the Supporting Information (SI). We solve the Kirchhoff equations to obtain the current as a function of the external perturbations *V̂* and Φ̂, and we obtain
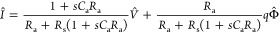
1As shown by Bertoluzzi et al.,^26^eq 1 can be compared to the general form
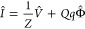
2At *V̂* = 0 we obtain the IMPS transfer function that corresponds to Figure 1b:
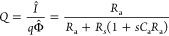
3The quantum efficiency is the fraction of the current obtained through the measuring wire, and it has the value^30^*Q*_0_ = *Q*(ω=0), namely
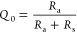
4The IMVS transfer function corresponds to the ratio of the open-circuit voltage to light flux at *I* = 0 in eq 2, with a minus sign:
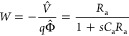
5The IS function occurs at *q*Φ̂ = 0 and according to eq 2 corresponds to the quotient *W*/*Q*:
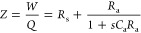
6

*The Transformation from Frequency to Time Domain.* Having obtained the different transfer functions of Figure 1a, we now consider the response of the system to a pulse of light, Φ(*t*) = Φ_0_Δ*t δ*(*t*), with δ(*t*) the Dirac delta function and Δ*t* the effective duration of the pulse, that contains an equivalent electrical charge θ_0_ = *q*Φ_0_Δ*t*. With this form of Φ(*t*), we are assuming that the light pulse is much faster than all internal relaxation modes of the system under consideration. By the inverse Laplace transform of *Q* we obtain the impulse response function,
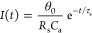
7where
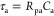
8
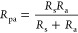
9

The photocurrent is shown in Figure 1e. It is related to the extraction of photogenerated charge *q*_extr_ that is not recombined at *R*_a_. In terms of the quantum efficiency, we obtain
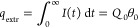
10

*Note about Time Scales.* In this paper, we show spectral shapes in frequency and time domains. We do not attach meaning to the numerical value of time constants; these can be highly variable depending on the specific system. Of course, the physical values are important. In practice, we find different spectral features in the transfer function plots, which means that a combination of decay types will occur in the time domain. The typical IMPS response shows features over many decades of frequency, from mHz to MHz.^7,31^ One can expect that there will be a variety of time transient decays depending on the time window that is measured, from ns to μs for bulk recombination phenomena^32^ to hours in the very slow depolarization of ions attached to the surface.^33^ The separation of spectral features in transfer functions is largely determined by the separation of the respective time constants. This factor determines if the features can be investigated separately in time and frequency domains. In this paper, we discuss different effects one by one, and the task of disentangling complex combined responses is left for experimental investigation of specific data sets.

*Negative Photocurrent Transients.* As mentioned earlier, negative features of TPC and TPV have been observed in many cases.^8−13^ These features have often been ascribed to nonequilibrium effects due to ionic polarization at the interfaces and its consequences. The appearance of a photovoltage creates an electrical field in the film that opposes the built-in field and modifies the amount and sign of charge in the double layer, producing internal currents that oppose the ordinary charge or discharge effect.^15^ The new conditions can also modify the rate of recombination at the interfaces. Here, we do not address such transient ionic effects, as we target the description of temporal decays that are obtained in a solar cell that operates in equilibrium conditions. We assume that there is a stationary equivalent circuit that decently describes the measurements of the frequency domain techniques in a reproducible fashion, without time drift effects.^34^

In this context we ask the question, can we obtain negative transients from a stationary steady state, arising from intrinsic properties of the system? A first possibility appears in the simple model of Figure 1 by choosing a negative resistance *R*_a_ and negative capacitance *C*_a_. Here we do not discuss the physical origin of these elements, but we note they are perfectly feasible.^28^ In addition, later in this paper we will show a diffusion model that presents negative characteristics in the *Q*-function.

In the model of Figure 1a with negative elements, we still obtain a positive relaxation time τ_a_ provided that *R*_s_ > −*R*_a_. Then, the *Q*-function appears in the third quadrant of the complex plane, as shown in Figure 1d, and the decay is a negative photocurrent due to the negative capacitance prefactor of eq 7, as indicated in Figure 1f.

*A Perovskite Solar Cell Model: RCRC Circuit.* A realistic equivalent circuit for a perovskite solar cell is shown in Figure 2. This model has been widely used to describe IS data,^2,35^ and the IMPS characteristics of this model have also been fully described.^7^ In the IS technique the model contains two arcs: the low-frequency arc is formed by *R*_1_ and the interfacial large capacitance *C*_1_, and the high-frequency arc contains *R*_3_ and the smaller geometric capacitance *C*_g_. The generalized response function is
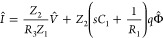
11*Z*_1_ and *Z*_2_ are given in the SI, where we also show how panels b–e of Figure 2 were determined. The small perturbation transfer functions are
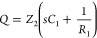
12
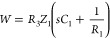
13

**Figure 2:**
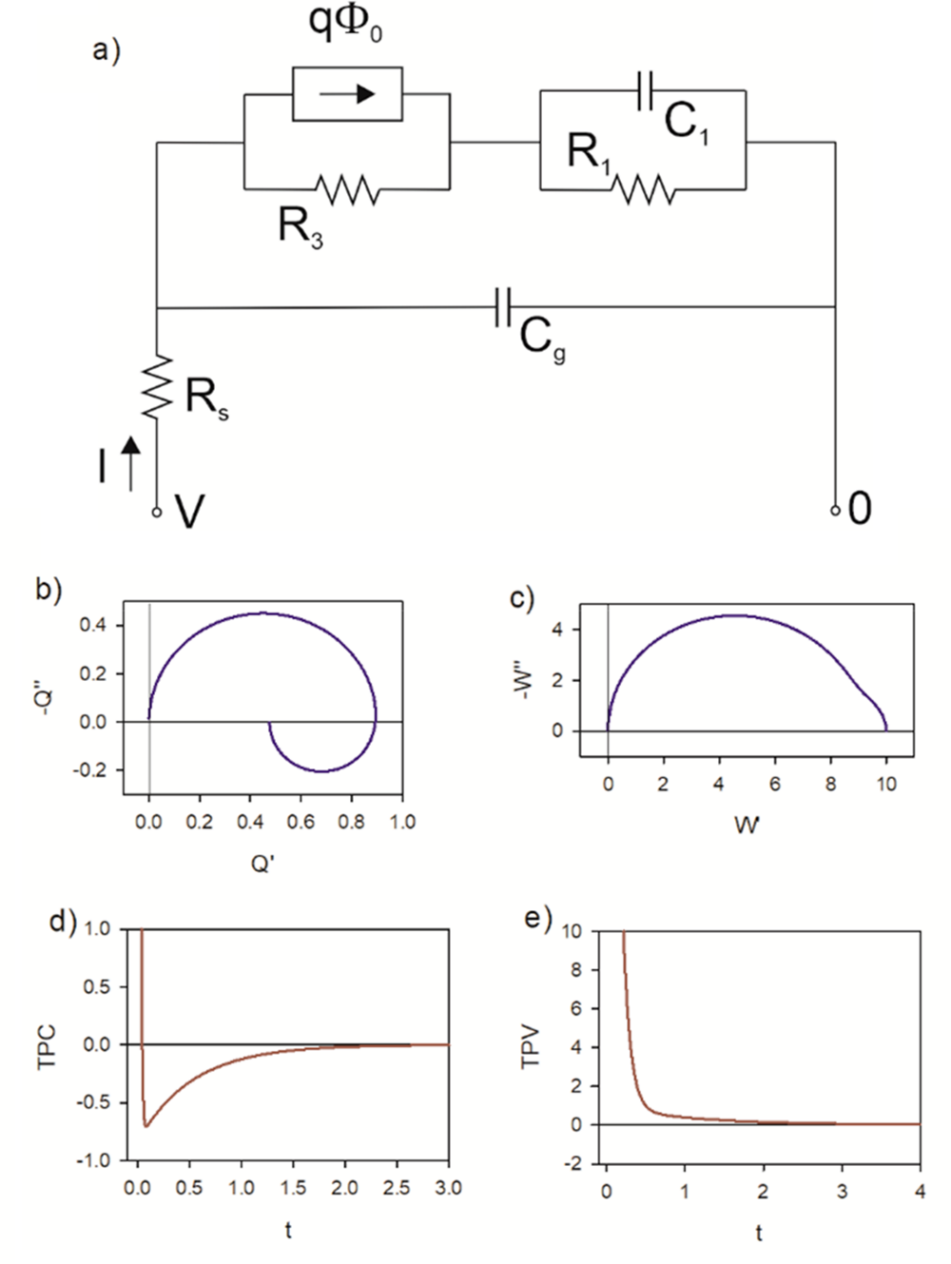
(a) Basic equivalent circuit of a perovskite solar cell. (b) IMPS, (c) IMVS, (d) TPC, and (e) TPV for the circuit with parameters *R*_s_ = 1 Ω, *R*_1_ = 10 Ω, *R*_3_ = 10, *C*_1_ = 0.1 F, and *C*_g_ = 0.01 F.

In Figure 2a it is observed that the generation source is in parallel to the high-frequency resistance. This crucial feature has been previously demonstrated experimentally.^4,5^ Due to the *R*_1_*C*_1_ subcircuit in series with the light source, the IMPS function shown in Figure 2b enters the negative part of the vertical −*Q*″ axis. This feature happens in the absence of negative capacitances. When the frequency decreases, the data first crosses the *Q*′ axis at the value
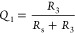
14but the negative arc reduces further *Q*′, and the quantum efficiency is^4^
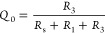
15

The transformation of *Q*(*s*) and *W*(*s*) to the time domain is described in the SI. In Figure 2d, we see that the TPC is composed of two parts. Initially, there is a regular positive decay corresponding to the positive arc at high frequencies in the complex-plane plot of *Q*. After this initial decay, there is a negative photocurrent spike due to the negative *Q*-arc that finally decays toward the origin. This property does not happen in TPV, which shows a regular decay corresponding to the fact that *W* remains in the first quadrant for all the values of frequency (Figure 2c,e).

*Generation–Diffusion–Recombination in the Frequency Domain.* The next and final case that we discuss is the generation–diffusion–recombination model applied for the PSC. The experimental arrangement to which the model applies is shown in Figure 3. The calculation of the transfer functions is based on the conservation equation governing the carrier density *n*(*x*,*t*),
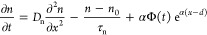
16Here, *D*_n_ is the diffusion coefficient, *n*_0_ the equilibrium density under dark conditions, τ_n_ the recombination lifetime, *d* the solar cell thickness, and α the light absorption coefficient. For both TPC and TPV we set the initial density to *n*(*x*,0) = *n*_0_ and the boundary at *x* = *d* to be reflecting electrons perfectly, giving the Neuman condition ∂_*x*_*n*(*x*,*t*)|_*x*=*d*_ = 0. In the case of TPV, the same Neuman condition applies to the boundary at *x* = 0; for TPC we assume perfect extraction of carriers at *x* = 0, yielding *n*(0,*t*) = *n*_0_.^36^ This generation–diffusion–recombination model has been applied to determining the diffusion coefficient of halide perovskites by TRPL.^20−22^ Related methods use direct imaging of the diffusing charges.^37^

**Figure 3:**
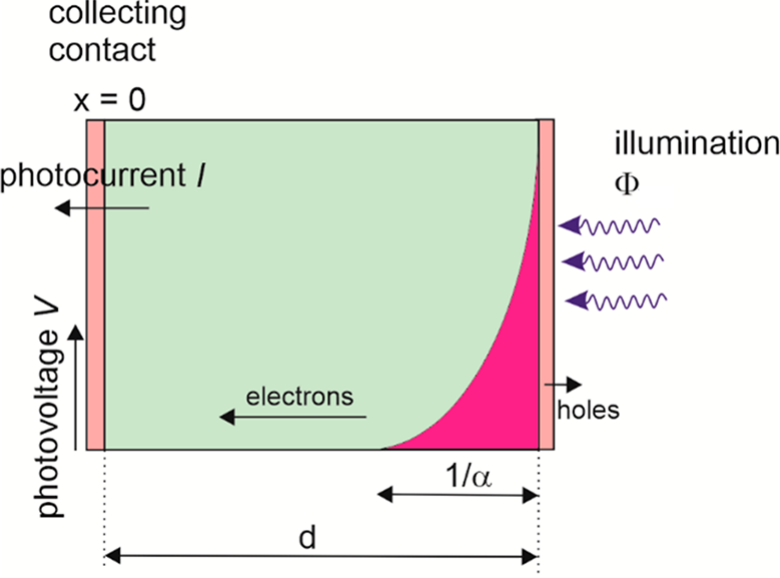
Scheme of the IMPS and IMVS measurement of a solar cell. Here the illuminated side (*x* = *d*) is the selective contact for holes, and the photogenerated electrons travel by diffusion to the other electrode (*x* = 0), where current and voltage are measured.

The problem of solving eq 16 in the frequency domain to obtain the IMPS transfer function has been fully described in ref (31). Here we summarize the properties of the solution. We express the *Q*-function in terms of the characteristic frequencies defined in Table 1. The three frequencies of Table 1 enable a full characterization of the possible different types of spectra, by the relationships indicated in Table 2, where we also make use of the electron diffusion length, defined as
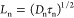
17

**Table 1:** Characteristic Frequencies of the Generation–Diffusion–Recombination System

ω_rec_ = τ_n_^–1^	recombination
ω_d_ = *D*_n_/*d*^2^	diffusion over the cell thickness *d*
ω_α_ = *D*_n_α^2^	diffusion over light absorption distance

**Table 2:** Spatial and Frequency Relations of the Generation–Diffusion–Recombination System

ω_d_/ω_rec_ = (*L*_n_/*d*)^2^	diffusion length to film size
ω_α_/ω_d_ = (*αd*)^2^	film size to light absorption length
ω_α_/ω_rec_ = (*αL*_n_)^2^	diffusion length to absorption length

The IMPS function has the expression^31^
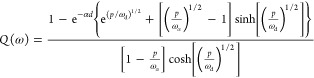
18
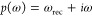
19For the case of IMVS (and TPV), we consider the relation of carrier density to the voltage at the selective contact for holes. For the Boltzmann statistics, we have that
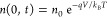
20

In eq 20 we consider that the voltage due to increasing electron density is negative. For a small perturbation of the voltage, we have
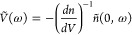
21With the definition of the chemical capacitance^29^
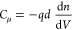
22we can rewrite eq 21 as
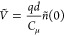
23Finally, the transfer function for IMVS has the form
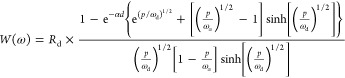
24where *R*_d_ is a diffusion resistance,
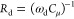
25

A comprehensive classification of spectra by the sets of parameters indicated in Table 3 is shown in Figure 4. The cases for a short penetration of the light are particularly interesting, as the spectra spiraling to the origin allow the determination of electron diffusion coefficient and lifetime as shown experimentally.^31^

**Table 3:** Values of Parameters Selected for Figure 4^a^

	Figure 4a	Figure 4b	Figure 4c	Figure 4d
*D*_n_	10^7^	10^5^	10^7^	10^5^
τ_n_	1.1 × 10^–4^	1.1 × 10^–4^	1.1 × 10^–4^	1.1 × 10^–4^
α	10/*d*	10/*d*	0.1/*d*	0.1/*d*
ω_rec_	9090	9090	9090	9090
ω_d_	10^5^	10^3^	10^5^	10^3^
ω_α_	10^7^	10^5^	10^3^	10
*L*_n_/*d*	3.31	0.331	3.31	0.331

a Distances in μm, time in s, *D*_n_ in μm^2^ s^–1^ (=10^6^ cm^2^ s^–1^), and frequencies in rad s^–1^. *d* = 10 μm and *R*_d_ = 1 Ω.

**Figure 4:**
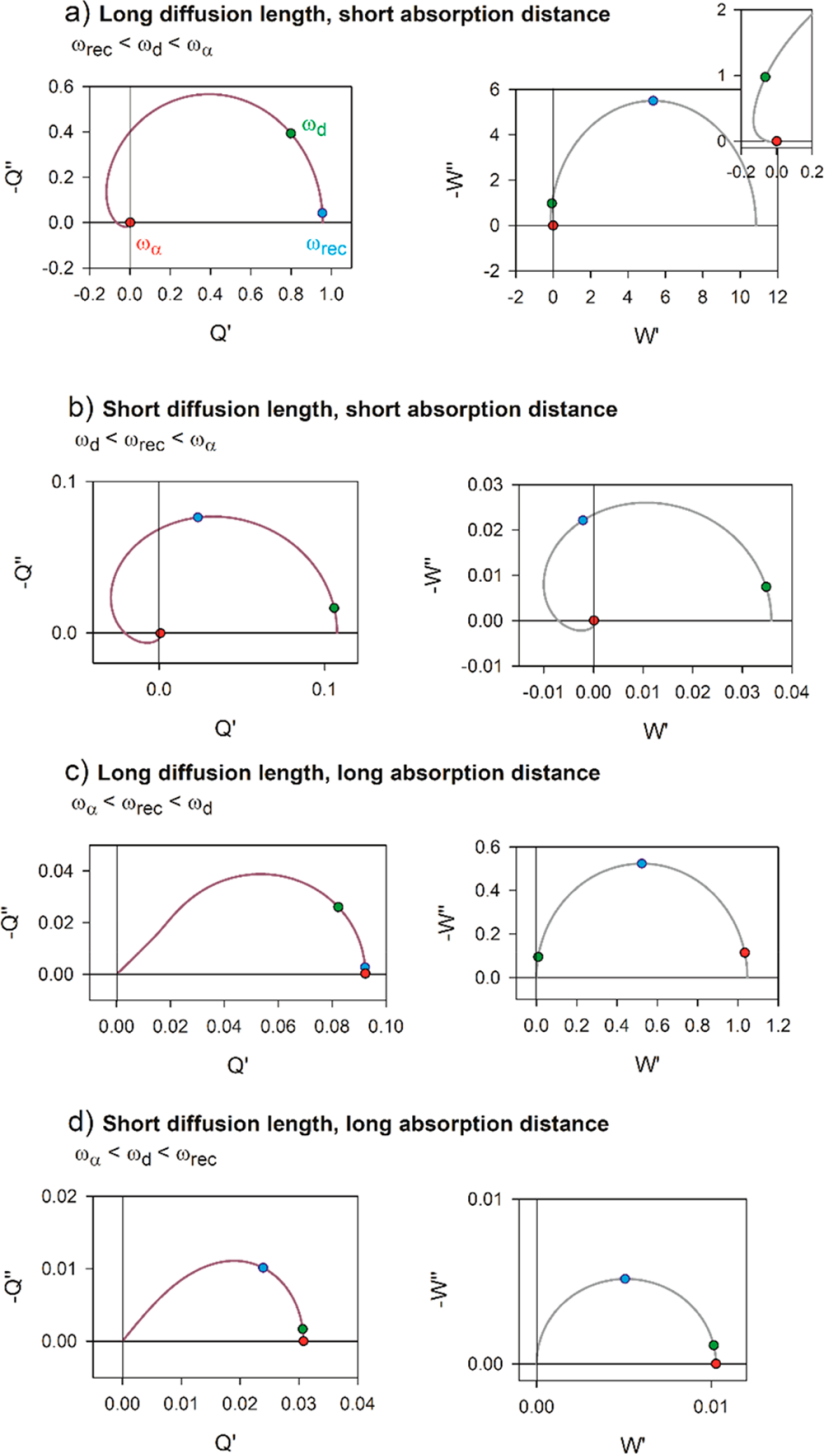
Representation of *Q* and *W* for the generation–diffusion–recombination model. For the used parameters, given in Table 3, the three characteristic frequencies of Table 1 take different values relative to each other.

*Transient Impulse Response of Generation–Diffusion–Recombination Model in the Time Domain.* In the SI, we obtained the TPC for a pulse of light *q*Φ(*t*) = θ_0_δ(*t*) in two ways. First, we solved eq 16 for the carrier density *n*(*x*,*t*) and obtained the TPC via *I*(*t*) = *qD*_n_∂_*x*_*n*(*x*,*t*)|_*x*=0_. Second, we performed an inverse Laplace transformation of the IMPS transfer function *Q*(ω) (eq 18). Both methods yield
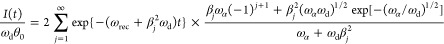
26where β_*j*_ = (*j* – 1/2)π and *j* = 1, 2, ... . We tested eq 26 against a numerical inverse Laplace transformation of *Q*(ω) and found excellent agreement (see SI).

For the analysis of experimental results, it is useful to obtain approximate expressions for *I*(*t*) at early and late times. At late times, only the *j* = 1 term contributes substantially to the above expression, such that
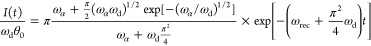
27For early times, we find, as shown in the SI, that the photocurrent decays as
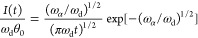
28where the *t*^–1/2^ scaling is characteristic of diffusion in a semi-infinite geometry.

Figure 5 presents plots of eqs 26 and 28 for several ω_rec_/ω_d_ and ω_α_/ω_d_. We observe that eq 28 accurately describes *I*(*t*) at early times and, accordingly, that *I*(*t*) does not depend on ω_rec_/ω_d_. Conversely, the behavior of *I*(*t*) at late times is chiefly determined by ω_rec_/ω_d_. Finally, we observe, for the large value ω_α_/ω_d_ = 100, that *I*(*t*) has a local maximum around ω_d_*t* = 0.2. This delayed peak in the TPC occurs because the electron charge initially created near the hole-selective contact is traveling across the sample and arrives at the electron extraction contact in competition with recombination through the film. It can be understood from the definition *I*(*t*) = *qD*_n_∂_*x*_*n*(*x*,*t*)|_*x*=0_, together with the carrier density *n*(*x*,*t*) (eq S8 in the SI), which we plot in Figure 6. Indeed, at ω_d_*t* = 0.1, *n*(*x*,*t*) is nonzero throughout the whole system, as opposed to the other times considered in Figure 6.

**Figure 5:**
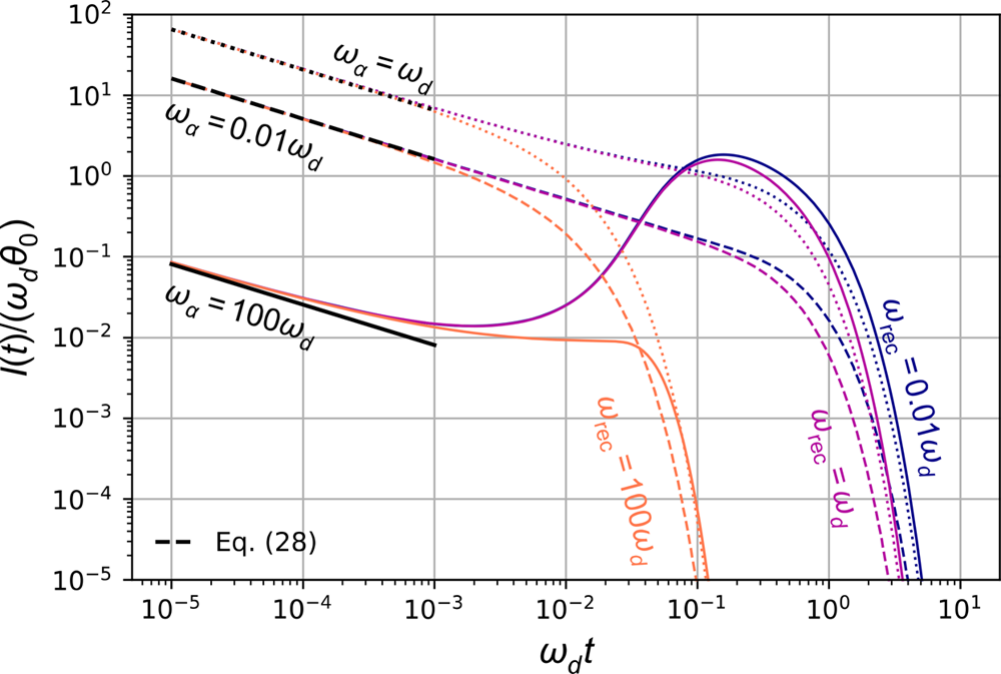
TPC (eq 27) for ω_rec_/ω_d_ = 0.01, 1, 100 (blue, purple, orange) and ω_α_/ω_d_ = 0.01, 1, 100 (dashed, dotted, solid). We also plot eq 28 in black for the same ω_α_/ω_d_ and corresponding patterns. We see that, for large ω_α_/ω_d_, meaning large α_d_ and hence a short adsorption length, there is a delayed peak in the photocurrent. This peak corresponds to the arrival of perturbations in *n*(*x*,*t*) near *x* = 0 (see Figure 6).

**Figure 6:**
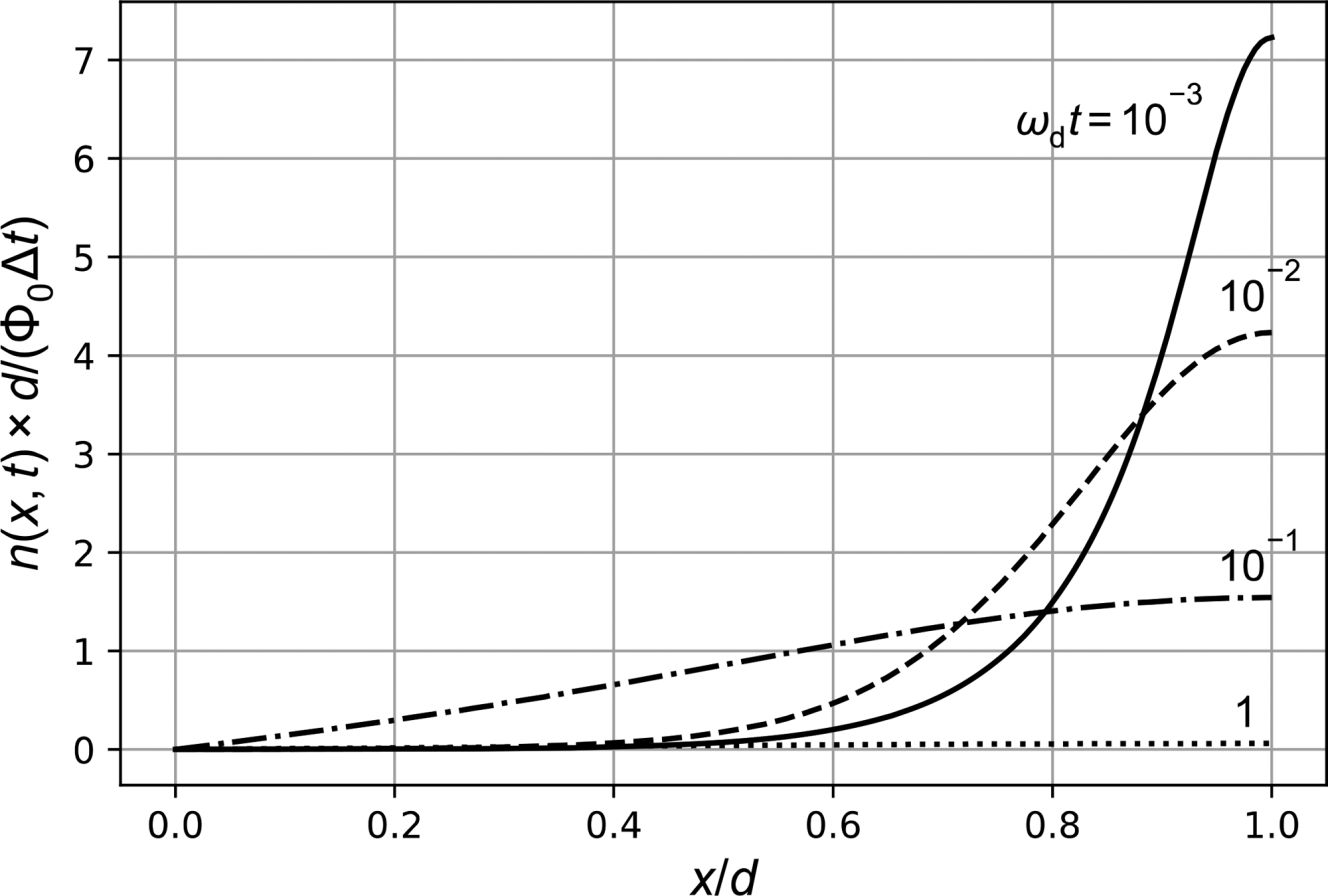
Photo density *n*(*x*,*t*) (eq S8 in the SI) for ω_rec_/ω_d_ = 1, ω_α_/ω_d_ = 100, and ω_d_*t* = 10^–3^, 10^–2^, 10^–1^, 1 (solid, dashed, dash-dotted, dotted). For ω_d_*t* = 10^–1^, we see that ∂*n*(*x*,*t*)/∂*x*|_*x*=0_ deviates substantially from zero, which explains the peak in the photocurrent *I*(*t*) = *qD*_n_∂*n*(*x*,*t*)/∂*x*|_*x*=0_ in Figure 5 around that time.

Next, we obtained the TPV for a pulse of light θ_0_δ(*t*), again in two ways as discussed in the SI. First, we again solved eq 16 for the carrier density *n*(*x*,*t*), now with a reflecting condition also at the other boundary (∂_*x*_*n*(*x*,*t*)|_*x*=0_ = 0), and obtained the TPV under the assumption of Boltzmann statistics (eq 20). Second, we performed an inverse Laplace transformation of the IMVS transfer function *W*(ω) (eq 24). Both methods yielded
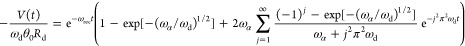
29As before, at late times, the above expression is dominated by the *j* = 1 term such that
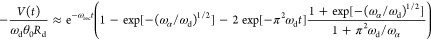
30Conversely, at *t* = 0, the sums in the above expression can be performed, yielding a constant photovoltage
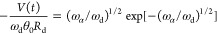
31

Figure 7 presents plots of eqs 29 and 31 for several ω_rec_/ω_d_ and ω_α_/ω_d_. We see there that *V*(*t*) shows an overshoot due to the late arrival of the photogenerated charges as in the case of the photocurrent.

**Figure 7:**
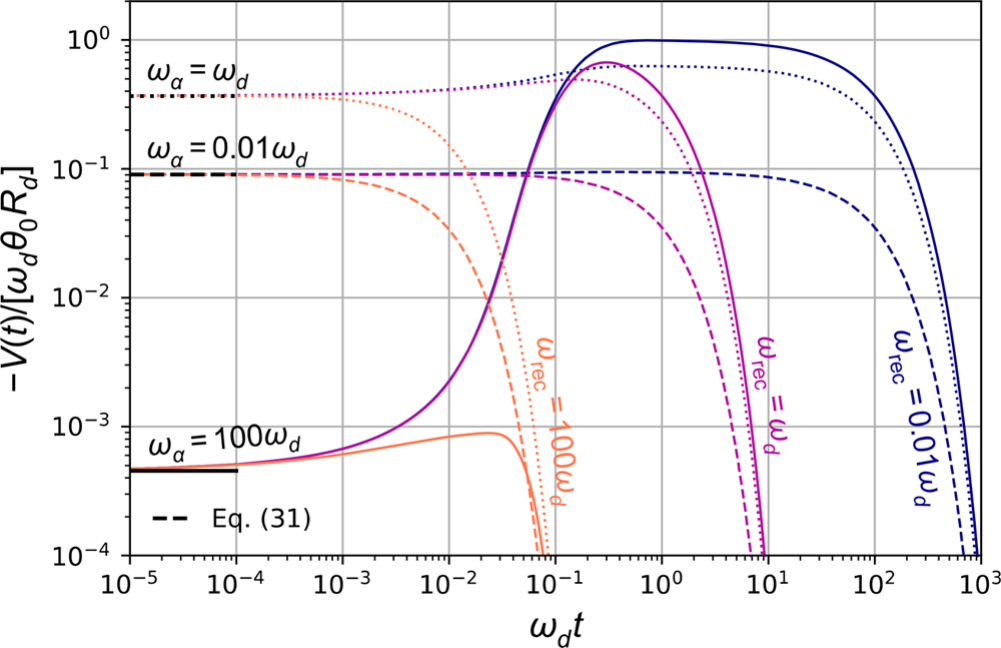
Plot of transient photovoltage (eq 29) for ω_rec_/ω_d_ = 0.01, 1, 100 (blue, purple, orange) and ω_α_/ω_d_ = 0.01, 1, 100 (dashed, dotted, solid). Also shown with black lines is the *t* = 0 expression (eq 31).

Finally, results for the OCVD method are presented in the SI. We show there that, after removing the illumination of a long-exposed solar cell, it relaxes with an ω_rec_- and ω_d_-dependent time scale, similar to what we found in eq 29.

*General Discussion and Conclusion.* For experimental studies, it may be necessary to use a combination of the different features analyzed in the previous models. For example, diffusion–recombination may replace the source term in Figure 1a, so that the spiraling feature would be affected by other elements in the equivalent circuit, such as the *R*_1_*C*_1_ negative loop of IMPS at low frequency and a modification of the high-frequency part by *R*_s_ and *C*_g_. The latter effect is usually denominated RC attenuation.^3,38,39^

In summary, we analyzed time- and frequency-domain measurement techniques that record a photocurrent or photovoltage in response to external stimuli. We have made manifest the connection of the impulse functions TPC and TPV to the respective light-modulated techniques in the frequency domain, IMPS and IMVS. We started from an elementary equivalent circuit and then described a realistic circuit for the description of measurements of a perovskite solar cell. Both models contain the possibility of negative photocurrent transients by operation in stationary conditions in which the cell is well stabilized. The analysis of diffusion and recombination shows spiraling spectra when the electrons are generated close to one contact and must travel across the sample. However, the presence of negative features in *Q* and *W* does not lead to negative transients in the time domain. The methods developed in this paper form a strong basis for the analysis of the complex temporal responses of perovskite solar cells.

## Supporting Information Available

Conventions for the sign of the transfer functions; impedance and transient behavior of the RCRC circuit; derivations of time transient decays of the generation–diffusion–recombination equation; and derivation of OCVD (PDF)
