# Supplementary material for: From Frequency Domain to Time Transient Methods for Halide Perovskite Solar Cells: The Connections of IMPS, IMVS, TPC, and TPV
Source: J Phys Chem Lett. 2021 Aug 13;12(33):7964–71. doi: 10.1021/acs.jpclett.1c02065 (PMC8404195; doi:10.1021/acs.jpclett.1c02065)
Supplement: Supplementary file 1 — jz1c02065_si_001.pdf [file jz1c02065_si_001.pdf]

Supporting Information to: From Frequency Domain to Time Transient Methods for Halide Perovskite Solar Cells: The Connections of IMPS, IMVS, TPC and TPV

Juan Bisquert[1*] and Mathijs Janssen[2*]

[1] Institute of Advanced Materials (INAM), Universitat Jaume I, 12006 Castelló, Spain

[2] Department of Mathematics, Mechanics Division, University of Oslo, N-0851 Oslo, Norway

Relationship between the transfer functions

The system is described by a relationship between three perturbations: voltage V, electrical current density I, and illumination flux Φ. current, voltage and photon flux. We consider small sinusoidal perturbations $\hat{V}(s)$, $\hat{I}(s)$ and $\hat{\Phi}(s)$ at a fixed angular frequency ω using the Laplace variable $s = i\omega$. We define the three transfer functions for impedance (Z), IMPS (Q), and IMVS (W).[1] In each case one of the perturbations is set to 0 as in the following table.

Table S1

Method	$\hat{I}$	$\hat{V}$	$q\hat{\Phi}$	Transfer function
IMPS		0		$Q = \hat{I}/q\hat{\Phi}$
IMVS	0			$W = -\hat{V}/q\hat{\Phi}$
IS			0	$Z = \hat{V}/\hat{I}$

The application of the three methods is shown in Fig. S1

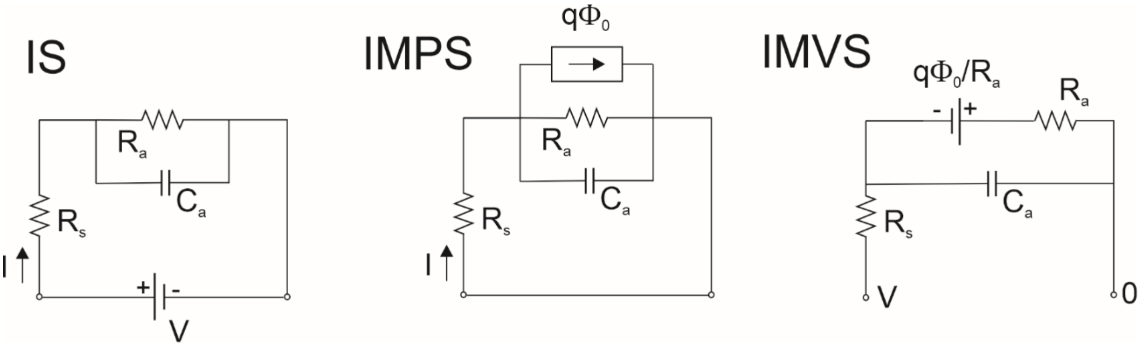

Fig. S1

Note that if the generation source $q\hat{\Phi}$ produces a positive current $\hat{I}$, then the photovoltage $\hat{V}$ is negative. The convention adopted is negative photopotential so that a minus sign occurs in W in Table S1. In this way the Re part of W is positive in the complex plane plot, what simplifies the representation.[2]

These three transfer functions are not independent but arise from the small perturbation of a general function. In general, by solving a physical model of a system with contacts for a small perturbation over a steady state, we obtain a relationship of the type

$$\hat{I} = F(\bar{V}, q\bar{\Phi})\hat{V} + G(\bar{V}, q\bar{\Phi})q\hat{\Phi} \quad (1)$$

From the Table S1 we can see that Eq. (1) can be written as[1]

$$\hat{I} = \frac{1}{Z}\hat{V} + Qq\hat{\Phi} \quad (2)$$

and

$$W = QZ \quad (3)$$

In conclusion, when we arrive at an equation of the type (1) we have found the transfer

functions since $Z = F^{-1}$ and $Q = G$.

One can get the same conclusion using derivatives, as $\hat{V}(s)$, $\hat{I}(s)$ and $\hat{\Phi}(s)$ are small perturbations. From Eq. (2)

$$Z = \left(\frac{\partial V}{\partial I}\right)_{\Phi} \tag{4}$$

$$Q = \frac{1}{q}\left(\frac{\partial I}{\partial \Phi}\right)_{V} \tag{5}$$

According to the convention in the above Table S1 we have

$$W = -\frac{1}{q}\left(\frac{\partial V}{\partial \Phi}\right)_{I} \tag{6}$$

We use the triple product rule

$$\left(\frac{\partial x}{\partial y}\right)_{z} = -\frac{\left(\frac{\partial z}{\partial y}\right)_{x}}{\left(\frac{\partial z}{\partial x}\right)_{y}} \tag{7}$$

which, for our variables, yields

$$\left(\frac{\partial V}{\partial I}\right)_{\Phi} = -\frac{\left(\frac{\partial \Phi}{\partial I}\right)_{V}}{\left(\frac{\partial \Phi}{\partial V}\right)_{I}} \tag{8}$$

In the above equation we identify $Z, Q,$ and W as

$$Z = \frac{qQ^{-1}}{qW^{-1}} = \frac{W}{Q} \tag{9}$$

If we had used an alternative definition for W,

$$W = \frac{1}{q}\left(\frac{\partial V}{\partial \Phi}\right)_{I} \tag{10}$$

we would have obtained

$$Z = -\frac{W}{Q} \tag{11}$$

References

(1) Bertoluzzi, L.; Bisquert, J. Investigating the Consistency of Models for Water Splitting Systems by Light and Voltage Modulated Techniques, *J. Phys. Chem. Lett.* **2017**, *8*, 172-180.

(2) Halme, J. Linking optical and electrical small amplitude perturbation techniques for dynamic performance characterization of dye solar cells, *Phys. Chem. Chem. Phys.* **2011**, *13*, 12435-12446.

R1 C1 R3 Cg model

```
Z1[w_] := (Cg I w + R3 Cg C1 (I w) ^2 + R3 Cg I w / R1 + C1 I w + 1 / R1) ^-1
Z2[w_] := (Rs / (R3 Z1[w]) + 1 / R3 + 1 / R1 + C1 I w) ^-1
Q[w_] := Z2[w] (C1 I w + 1 / R1)
W[w_] := R3 Z1[w] (C1 I w + 1 / R1)

Rs = 1;
R1 = 10;
R3 = 10;
Cg = 0.01;
C1 = 0.1; ty1 = ParametricPlot[{Re[W[2 π 10^pote]], -Im[W[2 π 10^pote]]},
  {pote, -8, 8}, PlotRange → {{-1, 1}, {-1, 1}},
  FrameLabel → {Style["Re W", FontWeight → "Bold"],
    Style["-Im W", FontWeight → "Bold"], "", ""}, Frame → True,
  FrameTicks → {{0, 1, 4, 6, 8, 10}, {0, 2, 4, 6, 8, 10}, None, None},
  PlotStyle → {{AbsoluteThickness[1]}}, AspectRatio → 1, ImageSize → 72 × 4`];

Show[ty1, PlotRange → {{-1, 1}, {-1, 1}}]
```

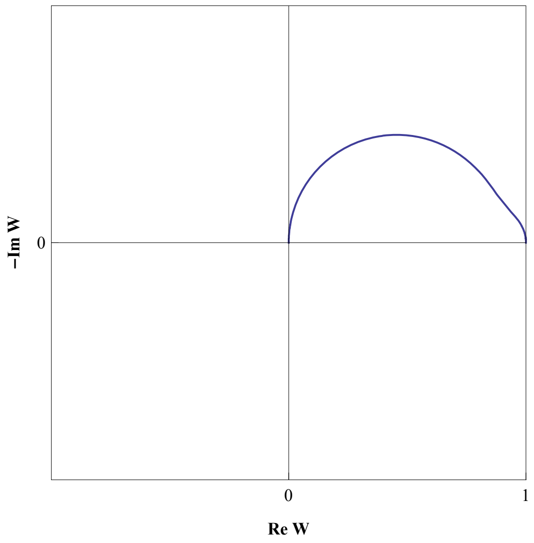


```
Z1R[w_] := (Cg w + R3 Cg C1 (w) ^2 + R3 Cg w / R1 + C1 w + 1 / R1) ^-1
WRC[w_] := R3 Z1R[w] (C1 w + 1 / R1)
```

```
mn = InverseLaplaceTransform[WRC[w], w , t]
FullSimplify[mn]
```

$$\left(e^{-\frac{\left(C1\,R1+Cg\,(R1+R3)+\sqrt{-4\,C1\,Cg\,R1\,R3+(C1\,R1+Cg\,(R1+R3))^2}\right)t}{2\,C1\,Cg\,R1\,R3}}\left(C1\,R1+Cg\,R1-Cg\,R3+e^{\frac{\sqrt{-4\,C1\,Cg\,R1\,R3+(C1\,R1+Cg\,(R1+R3))^2}\;t}{C1\,Cg\,R1\,R3}}\left(-(C1+Cg)\,R1+Cg\,R3\right)+\sqrt{-4\,C1\,Cg\,R1\,R3+(C1\,R1+Cg\,(R1+R3))^2}+e^{\frac{\sqrt{-4\,C1\,Cg\,R1\,R3+(C1\,R1+Cg\,(R1+R3))^2}\;t}{C1\,Cg\,R1\,R3}}\sqrt{-4\,C1\,Cg\,R1\,R3+(C1\,R1+Cg\,(R1+R3))^2}\right)\right)\Big/\left(2\,Cg\sqrt{-4\,C1\,Cg\,R1\,R3+(C1\,R1+Cg\,(R1+R3))^2}\right)$$

```
TPC[t_] :=
 10 (9.9029033784546` ⅇ^(-11.099019513592783` t) + 0.09709662154539923` ⅇ^(-0.9009804864072152` t))
```

```
tm1 = ParametricPlot[{uvi, TPC[uvi]},
   {uvi, 0, 3}, PlotRange → {{0, 2}, {-2, 30}}, FrameLabel →
    {Style["u", FontWeight → "Bold"], Style["I", FontWeight → "Bold"], "", ""},
   Frame → True, FrameTicks → {{0, 0.5, 1, 1.5, 2}, {0, 1}, None, None},
   PlotStyle → {{AbsoluteThickness[2], Blue}},
   AspectRatio → 0.5, ImageSize → 100 × 4`];
Show[tm1, PlotRange → {{0, 3}, {-1, 10}}]
```

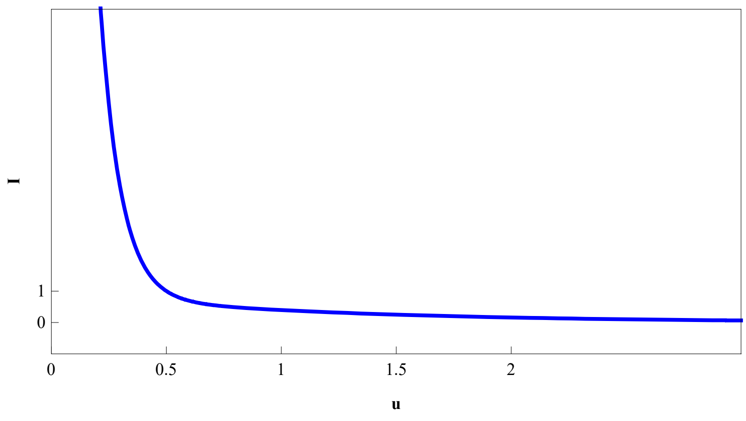

```
Rs = 1;
R1 = 10;
R3 = 10;
Cg = 0.01;
C1 = 0.1; ty1 = ParametricPlot[{Re[Q[2 π 10^pote]], -Im[Q[2 π 10^pote]]}, {pote, -8, 8},
  PlotRange → {{-1, 1}, {-1, 1}}, FrameLabel → {Style["Re Q", FontWeight → "Bold"],
    Style["-Im Q", FontWeight → "Bold"], "", ""}, Frame → True,
  FrameTicks → {{0, 1, 4, 6, 8, 10}, {0, 1, 4, 6, 8, 10}, None, None},
  PlotStyle → {{AbsoluteThickness[1]}}, AspectRatio → 1, ImageSize → 72 × 4`];

Show[ty1, PlotRange → {{-1, 1}, {-1, 1}}]
```

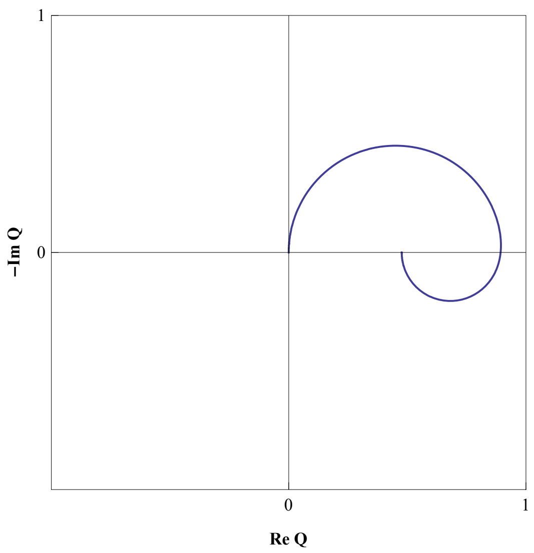


```
N[R3 / (Rs + R3)]
R3 / (Rs + R1 + R3)
```

0.909091

$\frac{10}{21}$

```
Z1R[w_] := (Cg w + R3 Cg C1 (w) ^2 + R3 Cg  w / R1  +  C1 w + 1 / R1) ^-1
Z2R[w_] := (Rs / (R3 Z1R[w]) + 1 / R3 + 1 / R1 + C1  w) ^-1
QRC[w_] := Z2R[w] (C1  w + 1 / R1)
```

```
mm = InverseLaplaceTransform[QRC[w], w, t]
FullSimplify[mm]
```

$$\left(e^{-\frac{(Cg\,(R1+R3)\,Rs+C1\,R1\,(R3+Rs))\,t}{2\,C1\,Cg\,R1\,R3\,Rs}}\left(\sqrt{Cg^2\,(R1+R3)^2\,Rs^2+C1^2\,R1^2\,(R3+Rs)^2-2\,C1\,Cg\,R1\,Rs\,(R1\,(R3-Rs)+R3\,(R3+Rs))}\;\mathrm{Cosh}\left[\frac{1}{2\,C1\,Cg\,R1\,R3\,Rs}\sqrt{Cg^2\,(R1+R3)^2\,Rs^2+C1^2\,R1^2\,(R3+Rs)^2-2\,C1\,Cg\,R1\,Rs\,(R1\,(R3-Rs)+R3\,(R3+Rs))}\;t\right]-(Cg\,(R1-R3)\,Rs+C1\,R1\,(R3+Rs))\,\mathrm{Sinh}\left[\frac{1}{2\,C1\,Cg\,R1\,R3\,Rs}\sqrt{Cg^2\,(R1+R3)^2\,Rs^2+C1^2\,R1^2\,(R3+Rs)^2-2\,C1\,Cg\,R1\,Rs\,(R1\,(R3-Rs)+R3\,(R3+Rs))}\;t\right]\right)\right)\Big/\left(Cg\,Rs\,\sqrt{Cg^2\,(R1+R3)^2\,Rs^2+C1^2\,R1^2\,(R3+Rs)^2-2\,C1\,Cg\,R1\,Rs\,(R1\,(R3-Rs)+R3\,(R3+Rs))}\right)$$

```
InverseLaplaceTransform[QRC[w], w, t]
```

$$100.839\,e^{-110.093\,t}-0.838828\,e^{-1.90749\,t}$$

```
TPC[t_] :=
 100.83882833194932` e^(-110.09251334519405` t) - 0.8388283319493264` e^(-1.9074866548059166` t)
```

```
TPC[0.1]
```

TPC[0.1]

```
tm1 = ParametricPlot[{uvi, TPC[uvi]},
   {uvi, 0, 3}, PlotRange → {{0, 2}, {-2, 30}}, FrameLabel →
    {Style["u", FontWeight → "Bold"], Style["I", FontWeight → "Bold"], "", ""},
   Frame → True, FrameTicks → {{0, 0.5, 1, 1.5, 2}, {0, 1}, None, None},
   PlotStyle → {{AbsoluteThickness[2], Blue}},
   AspectRatio → 0.5, ImageSize → 100 × 4`];
```

```
Show[tm1, PlotRange → {{0, 3}, {-1, 1.1}}]
```

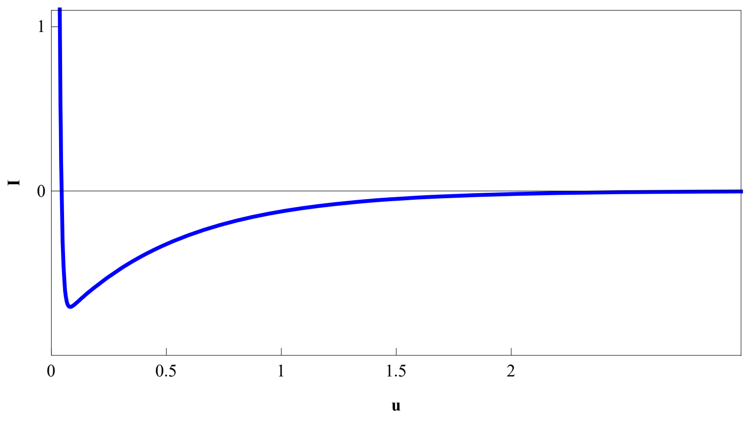

I. TRANSIENT PHOTOCURRENT (TPC) AFTER SPIKE ILLUMINATION

A. Generation-diffusion-recombination equation

We are interested in the carrier density $n(x,t)$ for $x \in [0,d]$ and $t \geq 0$, governed by Eq. (16) of the main text

$$\partial_t n = D_n \partial_x^2 n - \tau_n^{-1}(n - n_0) + G(x,t)\,, \tag{S1a}$$
$$n(x,0) = n_0\,, \tag{S1b}$$
$$n(0,t) = n_0\,, \tag{S1c}$$
$$\partial_x n(x,t)|_{x=d} = 0\,, \tag{S1d}$$

with D_n the diffusion constant, τ_n the recombination time, α the absorption constant, n_0 the photo density under dark conditions. Moreover, $G(x,t) = \alpha\phi_0(t)\exp[\alpha(x-d)]$ is the source term of electrons due to illumination; we will mostly consider an illumination spike $\Phi(t) = \Phi_0 \Delta t \delta(t)$, with $\delta(t)$ the Dirac delta function and Δt the effective duration of the pulse.

We simplify Eq. (S1) by substituting $n(x,t) = n_0 + T(x,t)\exp(-t/\tau_n)$ and multiplying by $\exp(t/\tau_n)$,

$$\partial_t T(x,t) = D_n \partial_x^2 T(x,t) + g(x,t)\,, \tag{S2a}$$
$$T(x,0) = 0\,, \tag{S2b}$$
$$T(0,t) = 0\,, \tag{S2c}$$
$$\partial_x T(x,t)|_{x=d} = 0\,, \tag{S2d}$$

where $g(x,t) \equiv G(x,t)\exp(t/\tau_n)$ represents the shifted source term. Equation (S2) corresponds to case X12 of the classification scheme of Ref. [1], where X means 1d cartesian, 1 refers to the Dirichlet condition, and 2 refers the Neumann condition. Reference [1] [Eq. (3.16)] gives the solution to Eq. (S2) as

$$T(x,t) = \int_0^t \mathrm{d}\tau \int_0^d \mathrm{d}x' g(x',\tau)\mathcal{G}_{X12}(x,t|x',\tau)\,, \tag{S3}$$

where $\mathcal{G}_{X12}(x,t|x',\tau)$ is the Green function corresponding to the X12 to geometry and bcs. Reference [1] gives two equivalent expressions for $\mathcal{G}_{X12}(x,t|x',\tau)$, the first of which [Eq. (X12.1)] converges fast at early times

$$\mathcal{G}_{X12}(x,t|x',\tau) = \frac{1}{\sqrt{4\pi D_n(t-\tau)}} \sum_{n=-\infty}^{\infty} (-1)^n \left\{ \exp\left[-\frac{(2nd + x - x')^2}{4D_n(t-\tau)}\right] - \exp\left[-\frac{(2nd + x + x')^2}{4D_n(t-\tau)}\right] \right\}\,, \tag{S4a}$$

and the second of which [Eq. (X12.8)] converges fast at late times,

$$\mathcal{G}_{X12}(x,t|x',\tau) = \frac{2}{d}\sum_{j=1}^{\infty} \exp\left[-\beta_j^2 \frac{D_n}{d^2}(t-\tau)\right] \sin\left(\beta_j \frac{x}{d}\right) \sin\left(\beta_j \frac{x'}{d}\right)\,, \tag{S4b}$$

where $\beta_j = \pi(j - 1/2)$, with $j = 1, 2, \ldots$. Below, we will use that $\cos\beta_j = \sin(2\beta_j) = 0$ and $\sin\beta_j = (-1)^{j+1}$.

With $n(x,t) = n_0 + T(x,t)\exp(-t/\tau_n)$ and inserting Eq. (S4b) into Eq. (S3) we find

$$n(x,t) = n_0 + e^{-t/\tau_n} \int_0^t \mathrm{d}\tau \int_0^d \mathrm{d}x' g(x',\tau) \mathcal{G}_{X12}(x,t|x',\tau) \tag{S5a}$$
$$= n_0 + \frac{2\alpha e^{-\alpha d}}{d} \sum_{j=1}^{\infty} \sin\left(\beta_j \frac{x}{d}\right) \int_0^t \Phi(\tau) \exp\left[\left(\frac{1}{\tau_n} + \beta_j^2 \frac{D_n}{d^2}\right)(\tau - t)\right] \mathrm{d}\tau \int_0^d e^{\alpha x'} \sin\left(\beta_j \frac{x'}{d}\right) \mathrm{d}x'\,. \tag{S5b}$$

Section 2.663 of Ref. [2] states that $\int \mathrm{d}x e^{ax} \sin(bx) = e^{ax}[a\sin(bx) - b\cos(bx)]/(a^2 + b^2)$. We thus find

$$\int_0^d e^{\alpha x'} \sin\left(\beta_j \frac{x'}{d}\right) \mathrm{d}x' = \frac{e^{\alpha d}\alpha \sin(\beta_j) - \cancel{e^{\alpha d}\cos(\beta_j)\beta_j/d} + \beta_j/d}{\alpha^2 + \beta_j^2/d^2} = d\frac{\alpha d e^{\alpha d}(-1)^{j+1} + \beta_j}{(\alpha d)^2 + \beta_j^2}\,. \tag{S6}$$

Next, we insert the above-mentioned $\Phi(t)$ into the t integral and find

$$\int_0^t \Phi_0(\tau) \exp\left[\left(\frac{1}{\tau_n} + \beta_j^2 \frac{D_n}{d^2}\right)(\tau - t)\right] \mathrm{d}\tau = \Phi_0 \Delta t \exp\left[-\left(\frac{1}{\tau_n} + \beta_j^2 \frac{D_n}{d^2}\right)t\right]\,. \tag{S7}$$

Inserting Eqs. (S6) and (S7) into Eq. (S5b) now gives

$$n(x,t) = n_0 + \frac{2\Phi_0 \Delta t}{d} \sum_{j=1}^{\infty} \sin\left(\beta_j \frac{x}{d}\right) \exp\left[-\left(\frac{1}{\tau_n} + \beta_j^2 \frac{D_n}{d^2}\right) t\right] \frac{(\alpha d)^2 (-1)^{j+1} + \beta_j \alpha d e^{-\alpha d}}{(\alpha d)^2 + \beta_j^2}\,, \tag{S8}$$

which is very similar to Eq. (S4) in the supplemental material of Ref. [3], who studied a related diffusion-recombination problem. From Eq. (S8) follows the photocurrent density $I(t) = qD_n \partial_x n(x,t)\big|_{x=0}$ at the collecting contact at $x = 0$ as

$$\frac{I(t)}{\omega_d q \Phi_0 \Delta t} = 2 \sum_{j=1}^{\infty} \exp\left[-\left(\frac{1}{\tau_n} + \beta_j^2 \frac{D_n}{d^2}\right) t\right] \frac{\beta_j (\alpha d)^2 (-1)^{j+1} + \beta_j^2 \alpha d e^{-\alpha d}}{(\alpha d)^2 + \beta_j^2}\,. \tag{S9}$$

We rewrite Eq. (S9) in terms of the frequencies $\omega_d = D_n/d^2$, $\omega_{\rm rec} = 1/\tau_n$, and $\omega_\alpha = D_n \alpha^2$ (and define $\theta_0 \equiv q\Phi_0 \Delta t$) as

$$\frac{I(t)}{\omega_d \theta_0} = 2 \sum_{j=1}^{\infty} \exp\left[-\left(\frac{\omega_{\rm rec}}{\omega_d} + \beta_j^2\right) \omega_d t\right] \frac{\beta_j (\omega_\alpha/\omega_d)(-1)^{j+1} + \beta_j^2 \sqrt{\omega_\alpha/\omega_d} \exp\left(-\sqrt{\omega_\alpha/\omega_d}\right)}{\omega_\alpha/\omega_d + \beta_j^2}\,, \tag{S10}$$

which shows that the solution is fully determined by two dimensionless parameters, $\omega_{\rm rec}/\omega_d$ and ω_α/ω_d. From Eq. (S10), one easily finds Eq. (26) of the main text.

To determine the early-time behaviour of $I(t)$, we re-evaluate Eq. (S5a), but now use Eq. (S4a) instead of Eq. (S4b). In the limit $t \to 0$, Eq. (S4a) is dominated by its $n = 0$ terms. We find, for $t \to 0$,

$$\begin{aligned} n(x,t) &= n_0 + \alpha \Phi_0 \Delta t e^{-t/\tau_n} \int_0^t \mathrm{d}\tau \int_0^d \mathrm{d}x' \delta(\tau) e^{\tau/\tau_n} \frac{\exp\left[\alpha(x'-d)\right]}{\sqrt{4\pi D_n (t-\tau)}} \left\{ \exp\left[-\frac{(x-x')^2}{4D_n(t-\tau)}\right] - \exp\left[-\frac{(x+x')^2}{4D_n(t-\tau)}\right] \right\} \\ &= n_0 + \frac{\alpha \Phi_0 \Delta t}{\sqrt{4\pi D_n t}} e^{-t/\tau_n} \int_0^d \mathrm{d}x' \exp\left[\alpha(x'-d)\right] \left\{ \exp\left[-\frac{(x-x')^2}{4D_n t}\right] - \exp\left[-\frac{(x+x')^2}{4D_n t}\right] \right\}\,. \end{aligned} \tag{S11}$$

We use Mathematica for the above integral and find

$$\begin{aligned} n(x,t) = n_0 + \frac{\alpha \Phi_0 \Delta t}{2} \exp\left(-\omega_{\rm rec} t + D_n t \alpha^2 - \alpha d\right) \Bigg\{ & e^{-\alpha x} \left[\mathrm{Erf}\left(\frac{x - 2D_n t \alpha}{\sqrt{4D_n t}}\right) - \mathrm{Erf}\left(\frac{x + d - 2D_n t\alpha}{\sqrt{4D_n t}}\right) \right] \\ & + e^{\alpha x} \left[\mathrm{Erf}\left(\frac{x + 2D_n t\alpha}{\sqrt{4D_n t}}\right) - \mathrm{Erf}\left(\frac{x - d + 2D_n t \alpha}{\sqrt{4D_n t}}\right) \right] \Bigg\}\,. \end{aligned} \tag{S12}$$

The photocurrent $I(t) = qD_n \partial_x n(x,t)\big|_{x=0}$ amounts to

$$\frac{I(t)}{\theta_0} = \alpha^2 D_n e^{-\omega_{\rm rec} t} \left[\frac{e^{-\alpha d} - e^{-d^2/(4D_n t)}}{\alpha \sqrt{\pi D_n t}} + \mathrm{Erf}\left(\sqrt{D_n t}\alpha\right) - \mathrm{Erf}\left(\frac{2D_n t \alpha - d}{\sqrt{4D_n t}}\right) \right]\,. \tag{S13}$$

To determine the dominant term at early times, we consider each term separately for $t \to 0$,

$$e^{-\omega_{\rm rec} t} \sim 1\,, \qquad \frac{e^{-\alpha d}}{\sqrt{\pi D_n t}} \propto \frac{1}{\sqrt{t}}\,, \qquad \frac{e^{-d^2/(4D_n t)}}{\sqrt{\pi D_n t}} \sim 0\,, \tag{S14a}$$

$$\mathrm{Erf}\left(\sqrt{D_n t}\alpha\right) \propto \sqrt{t}\,, \qquad \mathrm{Erf}\left(\frac{2D_n t\alpha - d}{\sqrt{4D_n t}}\right) \sim \mathrm{Erf}\left(\frac{-d}{\sqrt{4D_n t}}\right) \sim -e^{-d^2/(4D_n t)} \frac{\sqrt{4D_n t}}{d} \sim 0\,. \tag{S14b}$$

Hence, we find that $e^{-\alpha d}/\sqrt{\pi D_n t}$ gives the dominant contribution to $I(t)$ for $t \to 0$:

$$\frac{I(t)}{\omega_d \theta_0} \approx \frac{\alpha d \exp\left(-\alpha d\right)}{\sqrt{\pi D_n t/d^2}} = \sqrt{\frac{\omega_\alpha}{\pi \omega_d^2 t}} \exp\left(-\sqrt{\frac{\omega_\alpha}{\omega_d}}\right)\,, \tag{S15}$$

which corresponds to Eq. (28) of the main text.

B. TPC from inverse Laplace transform of IMPS

We write the Laplace transformation of a time-dependent function $f(t)$ as $\hat{f}(s) = \mathcal{L}\{f\}(s) = \int_0^\infty f(t)e^{-st}dt$. We also use the dimensionless $\bar{t} = \omega_d t$ and its conjugate variable $\bar{s} = s/\omega_d$. From the definition $\hat{Q}(s) = \hat{I}(s)/[q\hat{\Phi}(s)]$ we find that the spike illumination $\Phi(t) = \Phi_0 \Delta t \delta(t)$ causes a TPC governed by

$$I(t) = \mathcal{L}^{-1}\left\{\hat{I}(s)\right\} = \mathcal{L}^{-1}\left\{q\hat{\Phi}(s)\hat{Q}(s)\right\} = \theta_0 \mathcal{L}^{-1}\left\{\hat{Q}(s)\right\}, \tag{S16}$$

where we used that $\hat{\Phi}(s) = \mathcal{L}\left\{\Phi_0 \Delta t \delta(t)\right\} = \Phi_0 \Delta t$. For $\hat{Q}(s)$, we identify $s = \mathrm{i}\,\omega$ in the IMPS function $Q(\omega)$, Eq. (18) of the main text. We write $m^2 \equiv n^2 + \bar{s}$ with $n^2 = \omega_{\mathrm{rec}}/\omega_d$, so that $m = \sqrt{p(\omega)/\omega_d}$, and find

$$\hat{Q}(s) = \frac{1 - \exp(-\alpha d)\left[\exp(m) + \left(\frac{m}{\alpha d} - 1\right)\sinh(m)\right]}{\left[1 - \left(\frac{m}{\alpha d}\right)^2\right]\cosh(m)}. \tag{S17}$$

Equation (S17) has poles at $m = \pm\alpha d$, where the residues, however, are zero. Next, there are poles at $\bar{s}_j = m_j^2 - n^2$ with $m_j = \pm\mathrm{i}\,(j - 1/2)\pi \equiv \pm\mathrm{i}\,\beta_j$, with $j = 1, 2\ldots$ and $\mathrm{i} = \sqrt{-1}$. To determine the residues of $\hat{Q}(s)$ around these poles, we follow Ref. [5] and write

$$\cosh(m) \overset{\bar{s}\to\bar{s}_j}{=} \left.\frac{\sinh m}{2m}\right|_{\bar{s}=\bar{s}_j} (\bar{s} - \bar{s}_j) \qquad \Rightarrow \frac{1}{\cosh(m)} \overset{\bar{s}\to\bar{s}_j}{=} \frac{2\,\mathrm{i}\,(-1)^j m_j}{\bar{s} - \bar{s}_j}, \tag{S18}$$

where we used $m(\bar{s}_j) = \pm m_j$ and $\sinh m_j = \mathrm{i}\,(-1)^{j+1}$. We now evaluate Eq. (S16) and find

$$\frac{I(t)}{\theta_0} = \sum_{j=1}^{\infty} \mathrm{Res}\left(\hat{Q}(s)\exp(\bar{s}\bar{t}), s_j\right)$$

$$= \sum_{j=1}^{\infty} \mathrm{Res}\left\{\frac{1 - \exp(-\alpha d)\left[\exp(m_j) + \left(m_j/(\alpha d) - 1\right)\sinh(m_j)\right]}{1 - (m_j)^2/(\alpha d)^2} \frac{2i(-1)^j m_j \exp(\bar{s}_j\bar{t})}{\bar{s} - \bar{s}_j}, s_j\right\}$$

$$\frac{I(t)}{\omega_d\theta_0} = 2(\alpha d)^2 \sum_{j=1}^{\infty} \frac{1 - \exp(-\alpha d)\left[\exp(\mathrm{i}\,\beta_j) + \left(\frac{\mathrm{i}\,\beta_j}{\alpha d} - 1\right)\sinh(\mathrm{i}\,\beta_j)\right]}{(\alpha d)^2 + \beta_j^2} (-1)^{j+1}\beta_j \exp\left[-\left(\beta_j^2 + \frac{\omega_{\mathrm{rec}}}{\omega_d}\right)\bar{t}\right]$$

$$= 2(\alpha d)^2 \sum_{j=1}^{\infty} \frac{1 - \exp(-\alpha d)\left[\mathrm{i}\,(-1)^{j+1} - \left(\frac{\beta_j}{\alpha d} + i\right)(-1)^{j+1}\right]}{(\alpha d)^2 + \beta_j^2} (-1)^{j+1}\beta_j \exp\left[-\left(\beta_j^2 + \frac{\omega_{\mathrm{rec}}}{\omega_d}\right)\bar{t}\right], \tag{S19}$$

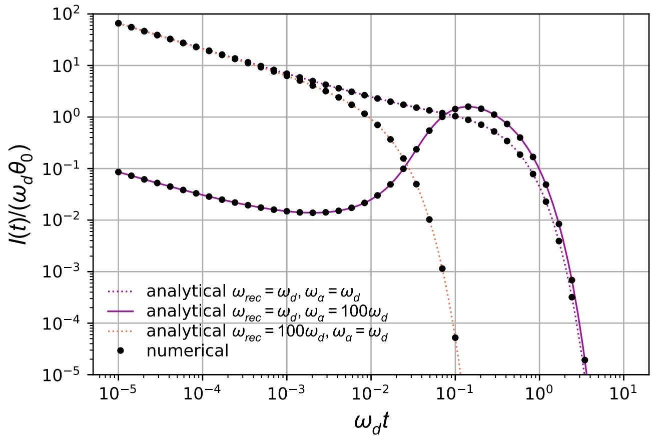

Figure S1. Comparison between the analytical result Eq. (S20) (lines) for the TPC and a numerical Laplace inversion of Eq. (S17) (dots), performed with the Talbot algorithm of the `mpmath` library [4].

where we used $\sin\beta_j = (-1)^{j+1}$ for the last line. We rewrite the last expression to

$$\frac{I(t)}{\omega_d\theta_0} = 2\sum_{j=1}^{\infty}\exp\left[-\left(\beta_j^2+\frac{\omega_{\rm rec}}{\omega_d}\right)\bar{t}\right]\frac{\beta_j(\alpha d)^2(-1)^{j+1}+\beta_j^2\alpha d e^{-\alpha d}}{(\alpha d)^2+\beta_j^2}\,, \tag{S20}$$

which, after inserting $\alpha d = \sqrt{\omega_\alpha/\omega_d}$ is identical to Eq. (S10). In Fig. S1, we compare Eq. (S20) with a numerical Laplace inversion of Eq. (S17) and find that they agree perfectly.

II. TRANSIENT PHOTOVOLTAGE (TPV) AFTER SPIKE ILLUMINATION

A. Generation-diffusion-recombination equation

To determine the TPV, we again study Eq. (S1), wherein we replace the absorbing boundary condition Eq. (S1c) by a reflecting boundary condition

$$\partial_x n(x,t)|_{x=0} = 0\,. \tag{S21}$$

The photodensity is now given by

$$n(x,t) = n_0 + e^{-t/\tau_n}\int_0^t \mathrm{d}\tau\int_0^d \mathrm{d}x' g(x',\tau)\mathcal{G}_{X22}(x,t|x',\tau)\,, \tag{S22}$$

with $g(x',\tau) = \alpha\Phi_0\Delta t\delta(\tau)\exp\left[\alpha(x'-d)\right]\exp(\tau/\tau_n)$ as before. Reference [1] again gives two equivalent expressions for $\mathcal{G}_{X22}(x,t|x',\tau)$, the first of which [Eq. (X22.1)] converges fast at early times

$$\mathcal{G}_{X22}(x,t|x',\tau) = \frac{1}{\sqrt{4\pi D_n(t-\tau)}}\sum_{n=-\infty}^{\infty}\left\{\exp\left[-\frac{(2nd+x-x')^2}{4D_n(t-\tau)}\right]-\exp\left[-\frac{(2nd+x+x')^2}{4D_n(t-\tau)}\right]\right\}\,, \tag{S23a}$$

and the second of which [Eq. (X22.3)] converges fast at late times,

$$\mathcal{G}_{X22}(x,t|x',\tau) = \frac{1}{d}+\frac{2}{d}\sum_{j=1}^{\infty}\exp\left[-j^2\pi^2\frac{D_n}{d^2}(t-\tau)\right]\cos\left(j\pi\frac{x}{d}\right)\cos\left(j\pi\frac{x'}{d}\right)\,. \tag{S23b}$$

We insert Eq. (S23b) into Eq. (S22) and find

$$\begin{aligned} n(x,t) &= n_0 + \frac{\alpha\Phi_0\Delta t}{d}e^{-t/\tau_n}\int_0^d \mathrm{d}x' e^{\alpha(x'-d)}\left[1+2\sum_{j=1}^{\infty}\exp\left[-j^2\pi^2\frac{D_n}{d^2}t\right]\cos\left(j\pi\frac{x}{d}\right)\cos\left(j\pi\frac{x'}{d}\right)\right] \\ &= n_0 + \frac{\Phi_0\Delta t}{d}e^{-t/\tau_n}\left[1-e^{-\alpha d}+2\alpha e^{-\alpha d}\sum_{j=1}^{\infty}\exp\left[-j^2\pi^2\frac{D_n}{d^2}t\right]\cos\left(j\pi\frac{x}{d}\right)\int_0^d \mathrm{d}x' e^{\alpha x'}\cos\left(j\pi\frac{x'}{d}\right)\right]\,, \end{aligned} \tag{S24}$$

Section 2.663 of Ref. [2] states that $\int \mathrm{d}x e^{ax}\cos(bx) = e^{ax}[a\cos(bx)+b\sin(bx)]/(a^2+b^2)$. We thus find

$$\int_0^d \exp(\alpha x')\cos\left(j\pi\frac{x'}{d}\right)\mathrm{d}x' = \frac{e^{\alpha d}\alpha\cos(j\pi)+e^{\alpha d}\sin(j\pi)\,j\pi/d-\alpha}{\alpha^2+j^2\pi^2/d^2} = \alpha d^2\frac{e^{\alpha d}(-1)^j-1}{(\alpha d)^2+j^2\pi^2}\,. \tag{S25}$$

Now, inserting Eq. (S25) into Eq. (S24) yields

$$n(x,t) = n_0 + \frac{\Phi_0\Delta t}{d}e^{-t/\tau_n}\left[1-e^{-\alpha d}+2\sum_{j=1}^{\infty}\exp\left(-j^2\pi^2\frac{D_n}{d^2}t\right)\cos\left(j\pi\frac{x}{d}\right)\frac{(-1)^j-e^{-\alpha d}}{1+j^2\pi^2/(\alpha d)^2}\right]\,. \tag{S26}$$

At the left ($x=0$) contact, this amounts to

$$\frac{n(0,t)}{n_0} = 1 + \frac{\Phi_0\Delta t e^{-t/\tau_n}}{n_0 d}\left[1-e^{-\alpha d}+2\sum_{j=1}^{\infty}\exp\left(-j^2\pi^2\frac{D_n}{d^2}t\right)\frac{(-1)^j-e^{-\alpha d}}{1+j^2\pi^2/(\alpha d)^2}\right]\,. \tag{S27}$$

From this we find the photovoltage as

$$-V(t) = \frac{k_B T}{q} \ln\left(\frac{n(0,t)}{n_0}\right) \approx \frac{k_B T}{q} \frac{\Phi_0 \Delta t}{n_0 d} e^{-t/\tau_n} \left[1 - e^{-\alpha d} + 2\sum_{j=1}^{\infty} \exp\left(-j^2\pi^2 \frac{D_n}{d^2} t\right) \frac{(-1)^j - e^{-\alpha d}}{1 + j^2\pi^2/(\alpha d)^2}\right], \tag{S28}$$

where the approximation holds for small illumination ($\Phi_0 \Delta t / n_0 d \ll 1$).

B. TPV from inverse Laplace transform of IMVS

We turn to determining the TPV from the IMVS transfer function $\hat{W}(s)$. From the definition $\hat{W}(s) = -\hat{V}(s)/[q\hat{\Phi}(s)]$ follows

$$V(t) = -\mathcal{L}^{-1}\left\{q\hat{\Phi}(s)\hat{W}(s)\right\} = -\theta_0 R_d \mathcal{L}^{-1}\left\{\frac{\hat{W}(s)}{R_d}\right\}. \tag{S29}$$

where we again used $\hat{\Phi}(s) = \mathcal{L}\left\{\Phi_0 \Delta t \delta(t)\right\} = \Phi_0 \Delta t$. Using the same notation as in Section I B, we rewrite the IMVS function $W(\omega)$, Eq. (24) of the main text, to

$$\frac{\hat{W}(s)}{R_d} = \frac{1 - \exp(-\alpha d)\left[\exp(m) + \left(\frac{m}{\alpha d} - 1\right)\sinh(m)\right]}{m\left[1 - \left(\frac{m}{\alpha d}\right)^2\right]\sinh(m)}. \tag{S30}$$

Equation (S30) has poles at $m = \pm\alpha d$, where the residues are again zero. Next, Eq. (S30) contains a pole at $\bar{s} = -n^2$. We use that

$$m \sinh m = m\left(m + \frac{m^3}{3!} + \ldots\right) = m^2\left(1 + \frac{m^2}{3!} + \ldots\right) = \left(n^2 + \bar{s}\right)\left(1 + \frac{m^2}{3!} + \ldots\right) \tag{S31}$$

to find the residue

$$\begin{aligned}
\mathrm{Res}\left(\frac{\hat{W}(s)}{R_d}\exp(\bar{s}\bar{t}), s = -n^2\omega_d\right) &= \omega_d \lim_{\bar{s}\to -n^2}\left(\bar{s} + n^2\right)\frac{\hat{W}(s)}{R_d} e^{\bar{s}\bar{t}} \\
&= \omega_d \frac{1 - \exp(-\alpha d)\left[\exp(0) + \left(\frac{0}{\alpha d} - 1\right)\sinh(0)\right]}{1 - (0)^2} e^{-n^2\bar{t}} \\
&= \omega_d\left(1 - e^{-\alpha d}\right) e^{-\frac{\omega_{\mathrm{rec}}}{\omega_d}\omega_d t}.
\end{aligned} \tag{S32}$$

Finally, there are poles at $\bar{s}_j = m_j^2 - n^2$ with $m_j = \pm \mathrm{i} j\pi$, where, again, $j = 1, 2\ldots$ and $\mathrm{i} = \sqrt{-1}$. We find that

$$\sinh(m) \overset{\bar{s}\to\bar{s}_j}{=} \left.\frac{\cosh m}{2m}\right|_{\bar{s}=\bar{s}_j}\left(\bar{s} - \bar{s}_j\right) \qquad \Rightarrow \frac{1}{\sinh(m)} \overset{\bar{s}\to\bar{s}_j}{=} \frac{2(-1)^j m_j}{\bar{s} - \bar{s}_j}, \tag{S33}$$

where we used $m(\bar{s}_j) = \pm m_j$, and $\cosh m_j = (-1)^j$. The residues of the poles at s_j amount to

$$\begin{aligned}
\sum_{j=1}^{\infty}\mathrm{Res}\left(\frac{\hat{W}(s)}{R_d}\exp(\bar{s}\bar{t}), s_j\right) &= \sum_{j=1}^{\infty}\mathrm{Res}\left\{\frac{1 - \exp(-\alpha d)\left[\exp(\mathrm{i}j\pi) + \cancel{\left(m_j/(\alpha d) - 1\right)\sinh(m_j)}\right]}{\cancel{m_j}\left[1 - (m_j)^2/(\alpha d)^2\right]}\frac{2(-1)^j \cancel{m_j}\exp(\bar{s}_j\bar{t})}{\bar{s} - \bar{s}_j}, s_j\right\} \\
&= 2\omega_d(\alpha d)^2\sum_{j=1}^{\infty}\frac{1 - \exp(-\alpha d)(-1)^j}{(\alpha d)^2 + j^2\pi^2}(-1)^j \exp\left[-\left(j^2\pi^2 + \frac{\omega_{\mathrm{rec}}}{\omega_d}\right)\omega_d t\right] \\
&= 2\omega_d(\alpha d)^2\sum_{j=1}^{\infty}\frac{(-1)^j - e^{-\alpha d}}{(\alpha d)^2 + j^2\pi^2}\exp\left[-\left(j^2\pi^2 + \frac{\omega_{\mathrm{rec}}}{\omega_d}\right)\omega_d t\right].
\end{aligned} \tag{S34}$$

We can now determine the TPV by evaluating Eq. (S29) with input from Eqs. (S32) and (S34),

$$-\frac{V(t)}{\omega_d\theta_0 R_d} = e^{-\omega_{\rm rec}t}\left[1-\exp\left(-\sqrt{\omega_\alpha/\omega_d}\right)+2\omega_\alpha\sum_{j=1}^{\infty}\frac{(-1)^j-\exp\left(-\sqrt{\omega_\alpha/\omega_d}\right)}{\omega_\alpha+j^2\pi^2\omega_d}\exp\left(-j^2\pi^2\omega_d t\right)\right], \tag{S35}$$

which corresponds to Eq. (29) of the main text. Using Eqs. (20), (22), and (25) of the main text, the prefactor in Eq. (S35) can be written to

$$\omega_d\theta_0 R_d = \cancel{\omega_d} q\Phi_0\Delta t\frac{1}{\cancel{\omega_d}C_\mu} = -\cancel{q}\Phi_0\Delta t\frac{1}{\cancel{q}d}\left(\frac{dn}{dV}\right)^{-1} = \frac{\Phi_0\Delta t}{d}\left(\frac{k_BT}{qn(0,t)}\right) \approx \frac{k_BT}{q}\frac{\Phi_0\Delta t}{n_0 d} \tag{S36}$$

where the last approximation holds only for small illumination.

At $t=0$, Eq. (S35) simplifies to Eq. (31) of the main text,

$$\begin{aligned}-\frac{V(t)}{\omega_d\theta_0 R_d} &= 1-\exp\left(-\sqrt{\omega_\alpha/\omega_d}\right)+2\sum_{j=1}^{\infty}\frac{(-1)^j}{1+j^2\pi^2\omega_d/\omega_\alpha}-2\exp\left(-\sqrt{\omega_\alpha/\omega_d}\right)\sum_{j=1}^{\infty}\frac{1}{1+j^2\pi^2\omega_d/\omega_\alpha}\\ &= 1-\exp\left(-\sqrt{\omega_\alpha/\omega_d}\right)+\left(\frac{\sqrt{\omega_\alpha/\omega_d}}{\sinh\sqrt{\omega_\alpha/\omega_d}}-1\right)-\exp\left(-\sqrt{\omega_\alpha/\omega_d}\right)\left(\frac{\sqrt{\omega_\alpha/\omega_d}}{\tanh\sqrt{\omega_\alpha/\omega_d}}-1\right)\\ &= \sqrt{\omega_\alpha/\omega_d}\exp\left(-\sqrt{\omega_\alpha/\omega_d}\right).\end{aligned} \tag{S37}$$

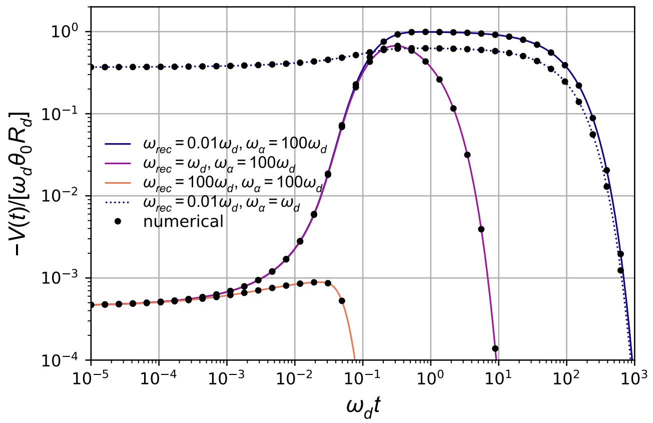

Figure S2. Comparison between the analytical result Eq. (S35) (lines) and a numerical Laplace inversion of Eq. (S30) (dots), performed with the Talbot algorithm of the `mpmath` library [4]

III. TPV AFTER SWITCHING ON A CONSTANT ILLUMINATION

A. Generation-diffusion-recombination equation

We solve the same equation as in the previous subsection, but now assume that at $t=0$, the light source is switched on and maintained—as opposed to the delta illumination considered before. The photodensity is again given by Eq. (S22), but we now consider $g(x',\tau)=\alpha\Phi_0\Theta(\tau)\exp\left[\alpha(x'-d)\right]\exp(\tau/\tau_n)$, with $\Theta(\tau)$ the Heaviside step function. We insert Eq. (S23b) into Eq. (S22) and find

$$n(x,t)=n_0+\alpha\Phi_0 e^{-t/\tau_n}\int_0^t \mathrm{d}\tau\int_0^d \mathrm{d}x' e^{\alpha(x'-d)}e^{\tau/\tau_n}\left\{\frac{1}{d}+\frac{2}{d}\sum_{j=1}^{\infty}\exp\left[-j^2\pi^2\frac{D_n}{d^2}(t-\tau)\right]\cos\left(j\pi\frac{x}{d}\right)\cos\left(j\pi\frac{x'}{d}\right)\right\}. \tag{S38}$$

The integral over $1/d$ easily gives

$$\frac{\alpha\Phi_0}{d}e^{-t/\tau_n}\int_0^t \mathrm{d}\tau e^{\tau/\tau_n}\int_0^d \mathrm{d}x' e^{\alpha(x'-d)} = \frac{\Phi_0\tau_n}{d}\left(1-e^{-t/\tau_n}\right)\left(1-e^{-\alpha d}\right) . \tag{S39}$$

The second term in Eq. (S38) involves the same x' integral as in Eq. (S25), and the following τ integral,

$$\int_0^t \mathrm{d}\tau \exp\left[\left(\frac{1}{\tau_n}+j^2\pi^2\frac{D_n}{d^2}\right)\tau\right] = \frac{1}{\frac{1}{\tau_n}+j^2\pi^2\frac{D_n}{d^2}}\left\{\exp\left[\left(\frac{1}{\tau_n}+j^2\pi^2\frac{D_n}{d^2}\right)t\right]-1\right\} . \tag{S40}$$

We can thus rewrite Eq. (S38) with Eqs. (S25), (S39) and (S40) to find

$$\frac{n(x,t)}{n_0} = 1+\frac{\Phi_0\tau_n}{n_0 d}\left(\left(1-e^{-t/\tau_n}\right)\left(1-e^{-\alpha d}\right)+\sum_{j=1}^{\infty}\frac{2\cos\left(j\pi x/d\right)}{1+j^2\pi^2\tau_n\frac{D_n}{d^2}}\frac{(-1)^j-e^{-\alpha d}}{1+\frac{j^2\pi^2}{(\alpha d)^2}}\left\{1-\exp\left[-\left(\omega_{rec}+j^2\pi^2\omega_d\right)t\right]\right\}\right) . \tag{S41}$$

Using Eq. (S41) we find the photovoltage $-V(t) = (k_BT/q)\ln(n(0,t)/n_0)$ as

$$-\frac{qV(t)}{k_BT} \approx \frac{\Phi_0\tau_n}{n_0 d}\left[\left(1-e^{-t/\tau_n}\right)\left(1-e^{-\sqrt{\omega_\alpha/\omega_d}}\right)+2\sum_{j=1}^{\infty}\frac{1-\exp\left[-\left(\omega_{rec}+j^2\pi^2\omega_d\right)t\right]}{1+j^2\pi^2\omega_d/\omega_{rec}}\frac{(-1)^j-\exp\left(-\sqrt{\omega_\alpha/\omega_d}\right)}{1+j^2\pi^2\omega_d/\omega_\alpha}\right] , \tag{S42}$$

where, again, the approximation holds for small illumination.

B. TPV from inverse Laplace transform of IMVS

Instead of a spike illumination, we now consider the TPV that is generated in response a step illumination, $\Phi(t) = \Phi_0\Theta(t)$, whose Laplace transform reads $\hat{\Phi}(s) = \Phi_0/s$. From the definition $\hat{W}(s) = -\hat{V}(s)/[q\hat{\Phi}(s)]$ now follows

$$V(t) = -\mathcal{L}^{-1}\left\{q\hat{\Phi}(s)\hat{W}(s)\right\} = -q\Phi_0 R_d\mathcal{L}^{-1}\left\{\frac{\hat{W}(s)}{sR_d}\right\} , \tag{S43}$$

For $\hat{W}(s)$, we again use Eq. (S30). First, the residues of Eq. (S30) at poles at $m = \pm\alpha d$ are again zero. Second, Eq. (S30) again contains a pole at $s = -n^2$. We find the residue to be

$$\begin{aligned}\mathrm{Res}\left(\frac{\hat{W}(s)}{sR_d}\exp(\bar{s}\bar{t}), s=-n^2\omega_d\right) &= \lim_{s\to -n^2\omega_d}\omega_d\left(\bar{s}+n^2\right)\frac{\hat{W}(s)}{sR_d}e^{\bar{s}\bar{t}} \\ &= \omega_d\frac{1-\exp\left(-\alpha d\right)\left[\exp\left(0\right)+\left(\frac{0}{\alpha d}-1\right)\sinh\left(0\right)\right]}{-n^2\omega_d[1-(0)^2]}e^{-n^2\bar{t}} \\ &= -\frac{\omega_d}{\omega_{\mathrm{rec}}}\left(1-e^{-\alpha d}\right)e^{-\omega_{\mathrm{rec}}t} .\end{aligned} \tag{S44}$$

Third, there is a pole at $s = 0$, which contributes the term

$$\begin{aligned}\mathrm{Res}\left(\frac{\hat{W}(s)}{sR_d}\exp(\bar{s}\bar{t}), s=0\right) &= \lim_{s\to 0}\frac{\hat{W}(s)}{R_d}e^{\bar{s}\bar{t}} \\ &= \frac{1-e^{-\alpha d}\left[\exp\left(n\right)+\left(\frac{n}{\alpha d}-1\right)\sinh\left(n\right)\right]}{n\left[1-\left(\frac{n}{\alpha d}\right)^2\right]\sinh\left(n\right)} = \frac{1-e^{-\alpha d}\left[\cosh\left(n\right)+\frac{n}{\alpha d}\sinh\left(n\right)\right]}{n\left[1-\left(\frac{n}{\alpha d}\right)^2\right]\sinh\left(n\right)} \\ &= \frac{\omega_d}{\omega_{\mathrm{rec}}}\frac{1-\exp\left(-\sqrt{\omega_\alpha/\omega_d}\right)\left[\cosh\left(\sqrt{\omega_{\mathrm{rec}}/\omega_d}\right)+\sqrt{\omega_{\mathrm{rec}}/\omega_\alpha}\sinh\left(\sqrt{\omega_{\mathrm{rec}}/\omega_d}\right)\right]}{\sqrt{\omega_d/\omega_{\mathrm{rec}}}\left[1-\frac{\omega_{\mathrm{rec}}}{\omega_\alpha}\right]\sinh\left(\sqrt{\omega_{\mathrm{rec}}/\omega_d}\right)}\end{aligned} \tag{S45}$$

Finally, there are again poles at $s_j = m_j^2 - n^2$ with $m_j = \pm \mathrm{i}\, j\pi$, with $j = 1, 2 \ldots$. We find

$$\sum_{j=1}^{\infty} \mathrm{Res}\left(\frac{\hat{W}(s)}{sR_d} \exp(\bar{s}\bar{t}), s_j \right) = \sum_{j=1}^{\infty} \mathrm{Res}\left\{ \frac{1-\exp(-\alpha d)\exp(\mathrm{i}\, j\pi)}{s\cancel{m_j}\left[1-(m_j)^2/(\alpha d)^2\right]} \frac{2(-1)^j \cancel{m_j} \exp(\bar{s}\bar{t})}{\bar{s}-\bar{s}_j}, s_j \right\}$$
$$= -2\omega_d(\alpha d)^2 \sum_{j=1}^{\infty} \frac{1}{j^2\pi^2\omega_d + \omega_{\mathrm{rec}}} \frac{1-\exp(-\alpha d)(-1)^j}{(\alpha d)^2 + j^2\pi^2} (-1)^j \exp\left[-\left(j^2\pi^2 + \frac{\omega_{\mathrm{rec}}}{\omega_d} \right) \omega_d t \right]$$
$$= -2\frac{\omega_d}{\omega_{\mathrm{rec}}} e^{-\omega_{\mathrm{rec}} t} \sum_{j=1}^{\infty} \frac{\exp\left(-j^2\pi^2\omega_d t\right)}{1+j^2\pi^2\omega_d/\omega_{\mathrm{rec}}} \frac{(-1)^j - e^{-\alpha d}}{1+j^2\pi^2/(\alpha d)^2} . \tag{S46}$$

Collecting terms, we find

$$-\frac{V(t)}{q\Phi_0 R_d}\frac{\omega_{\mathrm{rec}}}{\omega_d} = \frac{1-\exp\left(-\sqrt{\omega_\alpha/\omega_d}\right)\left[\cosh\left(\sqrt{\omega_{\mathrm{rec}}/\omega_d}\right) + \sqrt{\omega_{\mathrm{rec}}/\omega_\alpha}\sinh\left(\sqrt{\omega_{\mathrm{rec}}/\omega_d}\right)\right]}{\sqrt{\omega_d/\omega_{\mathrm{rec}}}\left[1-\frac{\omega_{\mathrm{rec}}}{\omega_\alpha}\right]\sinh\left(\sqrt{\omega_{\mathrm{rec}}/\omega_d}\right)}$$
$$- e^{-\omega_{\mathrm{rec}} t}\left[1-\exp\left(-\sqrt{\omega_\alpha/\omega_d}\right) + 2\sum_{j=1}^{\infty} \frac{\exp\left(-j^2\pi^2\omega_d t\right)}{1+j^2\pi^2\omega_d/\omega_{\mathrm{rec}}} \frac{(-1)^j - \exp\left(-\sqrt{\omega_\alpha/\omega_d}\right)}{1+j^2\pi^2\omega_d/\omega_\alpha}\right] . \tag{S47}$$

Similar to Eq. (S36), we can rewrite the prefactor of Eq. (S47) to

$$\omega_d \Phi_0 R_d \frac{\omega_d}{\omega_{\mathrm{rec}}} \approx \frac{k_B T}{q} \frac{\Phi_0}{n_0 d \omega_{\mathrm{rec}}} \tag{S48}$$

With Eq. (S48), we then see that the prefactor of Eq. (S42) corresponds to the one of Eq. (S47). The equivalence of their respective steady state limits is not obvious. Yet, Fig. S3 shows that Eqs. (S42) and (S47) coincide, and so does a numerical Laplace inversion of Eq. (S43). Comparing the expressions in Eqs. (S42) and (S47), we see that they are identical up to different steady state term: The steady-state contribution in Eq. (S42), inside the infinite sum, could still be identical to the steady-state in Eq. (S47), but we have no analytical derivation of this identity.

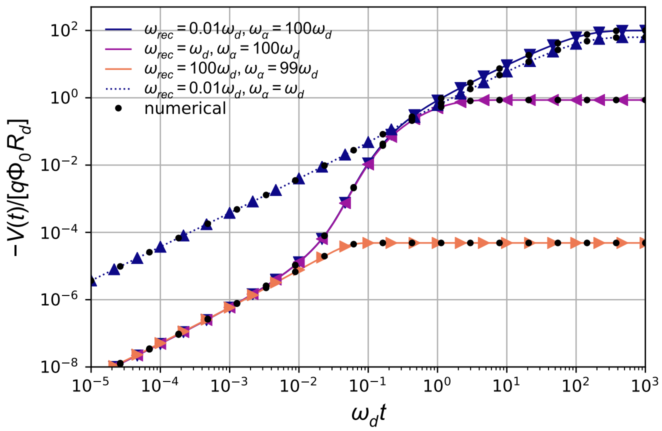

Figure S3. Photovoltage in response to a step illumination (without a step back). Comparison between a numerical Laplace inversion of Eq. (S43) (black dots), analytical result Eq. (S47) (symbols) and the solution to the diffusion equation Eq. (S42) (coloured lines). Because Eq. (S47) cannot handle cases for which $\omega_{\mathrm{rec}} = \omega_\alpha$, we choose the parameter settings for the orange line slightly different than normally. Equations (S42) and (S43) do not have this problem: they correspond well, also for $\omega_{\mathrm{rec}} = \omega_\alpha$.

IV. TPV DECAY AFTER REMOVING STEADY-STATE ILLUMINATION

We now calculate how the solar cell relaxes after removing the light source after a long period of illumination. Upon long constant illumination, the photodensity [Eq. (S41)] reaches the following steady state,

$$n_{s.s.}(x) = n_0 + \frac{\Phi_0\tau_n}{d}\left(1-e^{-\alpha d}\right) + \frac{2\Phi_0\tau_n}{d}\sum_{j=1}^{\infty}\frac{\cos\left(j\pi x/d\right)}{1+j^2\pi^2\tau_n\frac{D_n}{d^2}}\frac{(-1)^j-e^{-\alpha d}}{1+j^2\pi^2/(\alpha d)^2} \tag{S49}$$

Upon removing the illumination, the photodensity is governed by the diffusion-recombination equation

$$\partial_t n = D_n\partial_x^2 n - \tau_n^{-1}(n-n_0)\,, \tag{S50a}$$
$$n(x,0) = n_{s.s.}(x)\,, \tag{S50b}$$
$$\partial_x n(x,t)|_{x=0} = 0\,, \tag{S50c}$$
$$\partial_x n(x,t)|_{x=d} = 0\,, \tag{S50d}$$

wherein now no generation term appears. We again use the scaling $n(x,t) = n_0 + T(x,t)\exp(-t/\tau_n)$ to find

$$\partial_t T(x,t) = D_n\partial_x^2 T(x,t)\,, \tag{S51a}$$
$$T(x,0) = n_{s.s.}(x) - n_0\,, \tag{S51b}$$
$$\partial_x T(x,t)|_{x=0} = 0\,, \tag{S51c}$$
$$\partial_x T(x,t)|_{x=d} = 0\,. \tag{S51d}$$

The solution to the above equation in terms of Green's functions reads [see Eq. (3.16) of Ref. [1]]

$$T(x,t) = \int_0^d \mathrm{d}x'\mathcal{G}_{X22}(x,t|x',0)T(x',0)\,. \tag{S52}$$

Hence,

$$n(x,t) = n_0 + e^{-t/\tau_n}\int_0^d \mathrm{d}x'\mathcal{G}_{X22}(x,t|x',0)\left[n_{s.s.}(x)-n_0\right]\,. \tag{S53}$$

Using Eqs. (S23b) and (S49), we find

$$\begin{aligned} n(x,t) &= n_0 + e^{-t/\tau_n}\int_0^d\left[\frac{1}{d}+\frac{2}{d}\sum_{j=1}^{\infty}\exp\left(-j^2\pi^2\frac{D_n}{d^2}t\right)\cos\left(j\pi\frac{x}{d}\right)\cos\left(j\pi\frac{x'}{d}\right)\right] \\ &\quad\times\left[\frac{\Phi_0\tau_n}{d}\left(1-e^{-\alpha d}\right)+\frac{2\Phi_0\tau_n}{d}\sum_{k=1}^{\infty}\frac{\cos\left(k\pi x'/d\right)}{1+k^2\pi^2\tau_n\frac{D_n}{d^2}}\frac{(-1)^k-e^{-\alpha d}}{1+k^2\pi^2/(\alpha d)^2}\right]\mathrm{d}x' \\ &\equiv n_0 + I_1 + I_2 + I_3 + I_4\,, \end{aligned} \tag{S54}$$

where, in the last step, we split the integral in the following four terms,

$$I_1 = e^{-t/\tau_n}\int_0^d \mathrm{d}x'\frac{1}{d}\frac{\Phi_0\tau_n}{d}\left(1-e^{-\alpha d}\right)\,, \tag{S55a}$$
$$I_2 = e^{-t/\tau_n}\int_0^d \mathrm{d}x'\frac{1}{d}\frac{2\Phi_0\tau_n}{d}\sum_{k=1}^{\infty}\frac{\cos\left(k\pi x'/d\right)}{1+k^2\pi^2\tau_n\frac{D_n}{d^2}}\frac{(-1)^k-e^{-\alpha d}}{1+k^2\pi^2/(\alpha d)^2}\,, \tag{S55b}$$
$$I_3 = e^{-t/\tau_n}\int_0^d \mathrm{d}x'\frac{2}{d}\sum_{j=1}^{\infty}\exp\left[-j^2\pi^2\frac{D_n}{d^2}t\right]\cos\left(j\pi\frac{x}{d}\right)\cos\left(j\pi\frac{x'}{d}\right)\frac{\Phi_0\tau_n}{d}\left(1-e^{-\alpha d}\right)\,, \tag{S55c}$$
$$I_4 = e^{-t/\tau_n}\int_0^d \mathrm{d}x'\frac{2}{d}\sum_{j=1}^{\infty}\exp\left[-j^2\pi^2\frac{D_n}{d^2}t\right]\cos\left(j\pi\frac{x}{d}\right)\cos\left(j\pi\frac{x'}{d}\right)\frac{2\Phi_0\tau_n}{d}\sum_{k=1}^{\infty}\frac{\cos\left(k\pi x'/d\right)}{1+k^2\pi^2\tau_n\frac{D_n}{d^2}}\frac{(-1)^k-e^{-\alpha d}}{1+k^2\pi^2/(\alpha d)^2}\,. \tag{S55d}$$

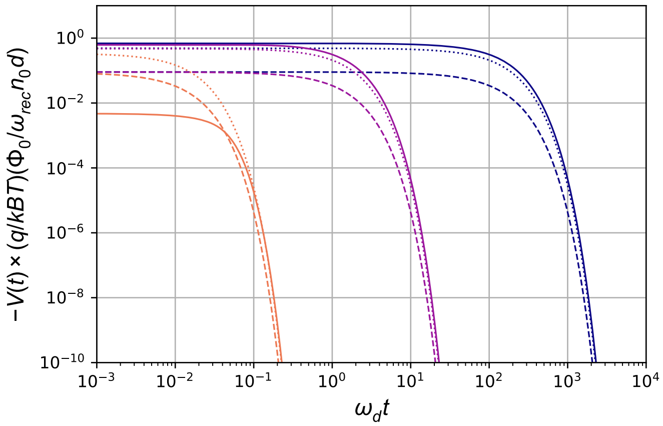

Figure S4. Equation (S58b): Photovoltage decay in response removing illumination, starting from a steady state. Colors and dashing correspond to ω_{rec}/ω_d and ω_α/ω_d as in the main text.

The first three expressions are easily evaluated: $I_1 = e^{-t/\tau_n} \frac{\Phi_0 \tau_n}{d} \left(1 - e^{-\alpha d}\right)$ and $I_2 = I_3 = 0$. Using the orthogonality of trigonometric functions, I_4 can be rewritten to

$$\begin{aligned} I_4 &= e^{-t/\tau_n} \frac{4\Phi_0 \tau_n}{d^2} \sum_{j,k=1}^{\infty} \exp\left[-j^2\pi^2 \frac{D_n}{d^2} t\right] \frac{\cos\left(j\pi \frac{x}{d}\right)}{1 + k^2\pi^2\tau_n \frac{D_n}{d^2}} \frac{(-1)^k - e^{-\alpha d}}{1 + k^2\pi^2/(\alpha d)^2} \int_0^d \mathrm{d}x' \cos\left(j\pi \frac{x'}{d}\right) \cos\left(k\pi \frac{x'}{d}\right) \\ &= e^{-t/\tau_n} \frac{2\Phi_0 \tau_n}{d} \sum_{j=1}^{\infty} \exp\left[-j^2\pi^2 \frac{D_n}{d^2} t\right] \frac{\cos\left(j\pi \frac{x}{d}\right)}{1 + j^2\pi^2\tau_n \frac{D_n}{d^2}} \frac{(-1)^j - e^{-\alpha d}}{1 + j^2\pi^2/(\alpha d)^2} . \end{aligned} \tag{S56}$$

Hence,

$$n(x,t) = n_0 + e^{-t/\tau_n} \frac{\Phi_0 \tau_n}{d} \left(1 - e^{-\alpha d}\right) + e^{-t/\tau_n} \frac{2\Phi_0 \tau_n}{d} \sum_{j=1}^{\infty} \exp\left(-j^2\pi^2 \frac{D_n}{d^2} t\right) \frac{\cos\left(j\pi \frac{x}{d}\right)}{1 + j^2\pi^2\tau_n \frac{D_n}{d^2}} \frac{(-1)^j - e^{-\alpha d}}{1 + j^2\pi^2/(\alpha d)^2} , \tag{S57}$$

which yields the decaying TPV as

$$\begin{aligned} -\frac{qV(t)}{k_B T} &= \ln\left(\frac{n(0,t)}{n_0}\right) \\ &= \ln\left(1 + \frac{\Phi_0 \tau_n}{n_0 d} e^{-\omega_{rec} t} \left[1 - \exp\left(-\sqrt{\omega_\alpha/\omega_d}\right) + 2\sum_{j=1}^{\infty} \frac{\exp\left[-j^2\pi^2\omega_d t\right]}{1 + j^2\pi^2\omega_d/\omega_{rec}} \frac{(-1)^j - \exp\left(-\sqrt{\omega_\alpha/\omega_d}\right)}{1 + j^2\pi^2\omega_d/\omega_\alpha}\right]\right) \end{aligned} \tag{S58a}$$

$$\approx \frac{\Phi_0 \tau_n}{n_0 d} e^{-\omega_{rec} t} \left[1 - \exp\left(-\sqrt{\omega_\alpha/\omega_d}\right) + 2\sum_{j=1}^{\infty} \frac{\exp\left[-j^2\pi^2\omega_d t\right]}{1 + j^2\pi^2\omega_d/\omega_{rec}} \frac{(-1)^j - \exp\left(-\sqrt{\omega_\alpha/\omega_d}\right)}{1 + j^2\pi^2\omega_d/\omega_\alpha}\right] . \tag{S58b}$$

---

[1] J. V. Beck, K. D. Cole, A. Haji-Sheikh, and B. Litkouhl, *Heat conduction using Green's function* (Taylor & Francis, 1992) p. 208-211.
[2] I. S. Gradshteyn and I. M. Ryzhik, *Table of integrals, series, and products* (Academic press, 2014).
[3] G. Xing, N. Mathews, S. Sun, S. S. Lim, Y. M. Lam, M. Grätzel, S. Mhaisalkar, and T. C. Sum, Science **342**, 344 (2013).
[4] F. Johansson *et al.*, *mpmath: a Python library for arbitrary-precision floating-point arithmetic (version 1.2.0)* (2021), `http://mpmath.org/`.
[5] M. Janssen and M. Bier, Phys. Rev. E **99**, 042136 (2019).
